# Pressure-tolerant Evolution in Rhodopsin of Deep-diving Whales

**DOI:** 10.1093/gbe/evag068

**Published:** 2026-03-16

**Authors:** Hayate Takeuchi, Takashi Hayakawa

**Affiliations:** Graduate School of Environmental Science, Hokkaido University, Sapporo, Hokkaido, Japan; Faculty of Environmental Earth Science, Hokkaido University, Sapporo, Hokkaido, Japan

**Keywords:** cetacean, codon substitution model, molecular dynamics, hydrostatic pressure, deep-sea, vision

## Abstract

Life in the deep sea presents extreme challenges to protein structure and function, with hydrostatic pressure serving as a significant source of molecular stress. Although cetacean rhodopsins have been thoroughly examined concerning their spectral tuning to the underwater light environment, their possible adaptations to pressure have yet to be explored. In this study, we investigated whether rhodopsin has undergone structural modifications that facilitate visual function during deep dives. Using a physicochemical property-based codon substitution model, we found that amino acid replacements associated with a radical shift in amino acid compressibility preferentially accumulated in deep-diving cetaceans belonging to the superfamily Physeteroidea and the family Ziphiidae. Molecular dynamics simulations further revealed that alanine at residue 299^7.46a^ confers enhanced pressure tolerance of rhodopsin relative to serine, as evidenced by lower isothermal compressibility, diminished flexibility, and reduced free-energy costs under high pressure. These findings identify residue 299^7.46a^ as a recurrent target for pressure adaptation in deep-diving cetaceans. More broadly, our study offers a novel perspective on cetacean visual adaptation, demonstrating that rhodopsins have evolved not only for spectral sensitivity but also for structural resilience under extreme hydrostatic pressure. This integrative framework, which combines evolutionary modeling with molecular dynamics simulations, advances our understanding of protein adaptation in the deep-sea environment.

Significance The adaptation of proteins to the extreme hydrostatic pressures encountered in the deep-sea remains considerably less understood than their adaptation to factors such as light or temperature. In this study, we demonstrated that residue 299^7.46a^ of cetacean rhodopsins is a recurrent target of molecular evolution and plays a pivotal role in conferring structural robustness against high pressure, with alanine at this position consistently reducing pressure-induced structural fluctuations and energetic costs compared to serine. These findings reveal that cetacean rhodopsins are influenced not only by optical constraints but also by the physical challenges imposed by pressure, addressing a long-standing gap in our understanding of sensory adaptation and establishing a framework for linking evolutionary signatures in amino acid sequences with their biophysical functions under extreme conditions.

## Introduction

Life in the deep-sea occurs under some of the most extreme environmental conditions on Earth. Situated beyond the reach of sunlight, organisms must navigate perpetual darkness while enduring immense hydrostatic pressures that increase by approximately one atmosphere every ten meters of depth (e.g. Somero 1992; Warrant and Locket 2004). In addition to these primary stressors, the deep ocean presents further challenges, including low temperatures and limited oxygen availability, which further constrain biological functions (e.g. Hourdez and Weber 2005; Yasuhara and Danovaro 2016). Among these stressors, hydrostatic pressure is particularly noteworthy due to its direct influence on molecular conformations and interactions, rendering it a compelling subject for understanding adaptation in the deep-sea environment. The structural robustness and free energy considerations of proteins under hydrostatic pressure have been examined in deep-sea archaea, bacteria, fish, and cephalopods, including rhodopsin, dihydrofolate reductase, α-actin, and lactate dehydrogenase (Medvedev et al. 2014; Wakai et al. 2014; Porter et al. 2016; Penhallurick et al. 2021; Maguire, Mercer, and Wiebe 2024).

Although the effects of hydrostatic pressure on protein structure and function have been investigated in diverse deep-sea taxa, comparable studies in aquatic mammals remain scarce. This gap is notable given that certain marine mammals undergo repeated and prolonged exposure to extreme pressures during deep dives. Among cetaceans, members of the odontocete superfamily Physeteroidea (including the families Physeteridae and Kogiidae) and the family Ziphiidae have developed remarkable capabilities for deep diving. These species regularly undertake dives exceeding 1,000 meters, with some recordings reaching approximately 3,000 meters (equivalent to a pressure of 30 MPa), placing them among the deepest-diving air-breathing animals (Baird et al. 2006; Watwood et al. 2006; Schorr et al. 2014). However, observations of Kogiidae are extremely limited due to their elusive behavior, and robust data on their diving depths remain scarce. Nevertheless, available evidence suggests that kogiids generally do not dive as deeply as physeterids or ziphiids, with an observation report coming from waters of approximately 1,400 meters depth (Baird 2005). It has been proposed that, despite their phylogenetic divergence, Physeteroidea and Ziphiidae have converged on similar diving adaptations through distinct evolutionary pathways (e.g. Lambert et al. 2015; Park et al. 2019). Prior research has emphasized molecular adaptations in these deep-diving lineages, particularly with regard to hypoxia tolerance during prolonged submergence (Tian et al. 2016; Isogai et al. 2018). Gaining an understanding of the molecular mechanisms underlying pressure tolerance in these deep-diving cetaceans provides a valuable insight into the constraints and innovations that influence vertebrate survival in the deep ocean.

Visual systems—reliant on the precise structural dynamics of light-sensitive proteins—must operate within the stringent constraints imposed by the deep ocean's combination of high hydrostatic pressure and limited light penetration. Rhodopsin, a G protein-coupled receptor (GPCR) integral to visual phototransduction, functions to detect light in dim-light environments (Sakmar et al. 2002). Animal opsins, including mammalian rhodopsin, are classified into four major groups: ciliary opsins (c-opsins), rhabdomeric opsins (r-opsins), Group-4 opsins, and xenopsins (De Vivo et al. 2025). Vertebrate rhodopsin belongs to the c-opsin clade, whereas most rhodopsins of invertebrates, including cephalopods, primarily use r-opsins for vision (De Vivo et al. 2025). Several studies have demonstrated that GPCRs, including rhodopsin, are highly vulnerable to conformational perturbations under elevated pressures (e.g. Somero 1992; Siebenaller and Murray 1999; Siebenaller and Garrett 2002). A previous study reported a negative correlation between habitat depth and the predicted adiabatic compressibility of visual opsins in deep-sea fishes and cephalopods (Porter et al. 2016), suggesting that opsins may evolve increased structural resilience under high hydrostatic pressure. Notably, these taxa rely on different opsin clades (vertebrate c-opsins versus cephalopod r-opsins), indicating that pressure-related trends may emerge across distinct opsin lineages. Given the essential role of rhodopsin in dim-light vision, it is reasonable to infer that maintaining its structural stability under extreme hydrostatic pressures is critical for visual function in deep-sea environments. Nonetheless, research on cetacean rhodopsins has predominantly focused on shifts in absorption spectra or retinal release rates as adaptive responses to the underwater light environment (e.g. Dungan and Chang 2017; Dungan and Chang 2022), leaving the potential molecular adaptations to hydrostatic pressure unexamined.

This study tested the hypothesis that structural adaptations in rhodopsin among deep-diving cetaceans enhance their tolerance to hydrostatic pressure. Evolutionary analyses using codon substitution models were employed to examine lineage-specific or site-specific trends in amino acid substitutions associated with decreased amino acid compressibility. Additionally, molecular dynamics simulations were conducted to assess the structural robustness and free energy of rhodopsin under simulated high-pressure conditions. Collectively, these methodologies offer complementary insights into the molecular mechanisms that enable the maintenance of visual performance within the high hydrostatic pressure environment of the deep-sea.

## Results

### Lineage- and Site-specific Patterns of Amino Acid Compressibility

To evaluate whether natural selection has influenced the structural properties of rhodopsin, we employed the codon substitution framework developed by Wong, Sainudiin, and Nielsen (2006), hereafter referred to as the WSN06 model. This model extends the standard Goldman and Yang (1994) model by categorizing nonsynonymous substitutions into conservative or radical variations based on a user-defined physicochemical property, as detailed in the Materials and Methods section. Within this framework, the instantaneous rates of conservative and radical nonsynonymous substitutions are scaled, relative to synonymous substitutions, by parameters *ω* and *γ*, respectively. In this investigation, amino acid compressibility (*K^0^*) was selected as the target physicochemical property, given its potential correlation with pressure-induced structural deformation, as indicated by Gromiha and Ponnuswamy (1993). Because an a priori biologically grounded cutoff between radical and conservative changes is difficult to define for *K^0^*, we evaluated a series of candidate partitions in which the top *P*% of nonsynonymous substitutions ranked by |Δ*K^0^*| were designated as radical (*P* = 10, 20, …, 90), and selected the best-fitting partition using likelihood-based model comparison (see Materials and Methods). Rhodopsin coding sequences were obtained from published genome assemblies of 51 cetacean species, along with *Hippopotamus amphibius*, a species considered among the most closely related extant taxa (Table S1).

**FIG. 1.**
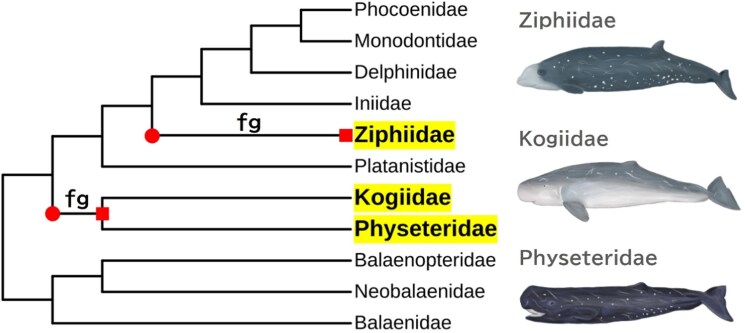
The phylogenetic tree of cetaceans at the family level is shown above. Branches designated as “fg” indicate foreground branches, for which we examined whether radical substitutions—defined as substitutions associated with substantial changes in compressibility (*K^0^*)—were favored in comparison to background branches. The ancestral states of *K^0^* at each codon site were inferred, and the differences in compressibility between nodes marked by red circles and those marked by red squares were analysed. Illustrated by Hayate Takeuchi.

For the branch model analysis, branches leading to Physeteroidea and Ziphiidae were designated as foreground (fg) branches. In contrast, all remaining branches were classified as background (bg) branches (see Fig. 1). An alternative model, which permits the separate estimation of *γ*_fg_ and *γ*_bg_, was compared to a null model that assumes a common *γ* across all branches, using the AIC comparison and likelihood ratio test (LRT). Among the candidate radical/conservative partitions, the model defining radical substitutions as those in the top 60% of |Δ*K^0^*| provided the best fit according to AIC (Table 1; Table S2). The log-likelihoods, AIC values, and parameter estimate for all candidate partitions are summarized in Table S2. Under the best-fitting partition, *γ*_fg_ was estimated as 0.415, whereas *γ*_bg_ was 0.201. The model allowing distinct *γ* values for foreground and background branches fit the data significantly better than the single-*γ* model (LRT, *P* < 0.005). This outcome indicates an elevated tendency for substitutions associated with larger *K^0^* changes along deep-diving lineages. The log-likelihood and AIC for the best-fitting partition model, as well as other parameter estimates including *κ*, *ω*, and *γ*, are provided in Table 1. The phylogenetic tree inferred under the best-fitting partition model is shown in Fig. S1.

**Table 1:** Log-likelihood (ℓ), AIC, and estimated parameters (*κ*, *ω*, *γ*) in the branch model

Model	ℓ	AIC	*κ*	*ω*	*γ*
Alternative	−3104.646	6419.292	6.044	0.769	*γ*_fg_ = 0.415, *γ*_bg_ = 0.201
Null	−3109.448	6426.896	6.048	0.766	0.215

Next, we applied a site model to identify codon-specific amino acid substitution patterns linked to changes in *K^0^*. Each codon was classified into four groups based on *ω* (≤1 or > 1) and *γ* (≤1 or > 1), testing for excess sites with *γ*_1_ > 1 (see Materials and Methods). Among the candidate radical/conservative partitions, the model defining radical substitutions as those in the top 60% of |Δ*K^0^*| provided the best fit according to AIC. The log-likelihoods, AIC values, and posterior probability of belonging to each site class for all candidate partitions are summarized in Table S3. Under the best-fitting partition, the alternative model, which permits all four site classes, demonstrated a significantly improved fit to the data in comparison to the null model, which only allowed site classes with *γ_0_* ≤ 1 (LRT, *P* < 0.0001). This outcome indicates that positive selection favoring amino acid substitutions linked to larger *K^0^* changes was operative at specific codon sites. The estimated proportions of sites assigned to each site class (see Materials and Methods for details on site classes) were 0.554326 for class (i), 0.177145 for class (ii), 0.176279 for class (iii), and 0.092250 for class (iv). Accordingly, a total of 26.9% of sites were inferred to belong to categories with *γ_1_* > 1 (site classes (ii) and (iv)), denoting an increased rate of substitutions associated with larger *K^0^* changes. Additionally, 24 codon sites with a posterior probability exceeding 0.95 for assignment to site classes with *γ_1_* > 1 are listed in supplementary Table S4.

To identify candidate sites involved in convergent structural adaptations of rhodopsin against high hydrostatic pressures, we reconstructed the ancestral states of *K^0^* at each codon site for the nodes leading to the families Physeteroidea and Ziphiidae (see Materials and Methods). Subsequently, we screened for sites that exhibited convergent amino acid substitutions that resulted in decreased *K^0^* in both deep-diving lineages. By integrating these results with the site model analysis, we were able to identify codon sites assigned to site classes with *γ_1_* > 1 that also underwent such convergent substitutions. This screening identified a single codon site, corresponding to the S299^7.46a^A substitution, as a candidate mutation potentially contributing to the structural robustness of rhodopsin under hydrostatic pressure in deep-diving cetaceans. The list of codon sites that independently underwent amino acid substitutions associated with decreased *K^0^* in each of the deep-diving lineages, Physeteroidea and Ziphiidae, respectively, is presented in Table 2.

**Table 2:** List of codon sites that independently underwent amino acid substitutions associated with decreased compressibility in Physeteroidea and Ziphiidae

Physeteroidea		Ziphiidae	
Substitution pattern^a^	Δ*K*^0,b^	Substitution pattern	Δ*K*^0^
CTG (L) 7 CCG (P)^c^ ATC (I) 290^7.36a^ GTC (V) TCC (S) 299^7.46a^ GCC (A)	−8.33 −1.13 −4.28	AAG (K) 16 CAC (H) TTT (F) 88^2.55a^ CTT (L) ATT (I) 189^45.52a^ GTT (V) CTC (L) 194 CCC (P) ACG (T) 195 TCG (S) ATC(I) 205^5.41a^ GTC (V) ATC(I) 255^6.38a^ GTC (V) TCC (S) 299^7.46a^ GCC (A)	−1.50 −2.75 −1.16 −8.45 −1.18 −1.16 −1.15 −4.30

^a^The codon state of the ancestral node is probabilistically defined. The most probable codon state is listed in the substitution pattern column.

^b^The substitution patterns are listed in the order of “from codon (amino acid), site, and the Ballesteros–Weinstein numbering scheme, to codon (amino acid)”.

^c^Δ*K*^0^ represents the degree of decrease in the absolute value of compressibility by amino acid substitutions that occurred in each deep-sea lineage.

### Contrasting Pressure Responses of A/S299^7.46a^ Rhodopsin Variants

To evaluate the influence of residue 299^7.46a^ on the structural stability of rhodopsin under hydrostatic pressure, molecular dynamics simulations were conducted under two pressure conditions: atmospheric (0.1 MPa) and elevated (30 MPa, equivalent to a water depth of 3,000 m), employing GROMACS v2025.1 (Abraham 2015). Rhodopsin models were constructed for two odontocete species with contrasting diving ecologies: the deep-diving Cuvier's beaked whale (*Ziphius cavirostris*) and the shallow-diving harbor porpoise (*Phocoena phocoena*). Each species was examined in its naturally occurring variant—alanine at position 299 in *Z. cavirostris* and serine in *P. phocoena*—as well as in reciprocal mutants bearing the alternative residue (A299S in *Z. cavirostris* and S299A in *P. phocoena*, respectively). As supplementary analyses to assess the robustness of the observed trends, we additionally simulated (i) two non-deep-diving cetaceans that retain A299 (baiji, *Lipotes vexillifer*, and pygmy right whale, *Caperea marginata*) using wild-type and A299S mutant models, and (ii) reconstructed ancestral rhodopsins for the nodes leading to Physeteroidea and Ziphiidae, respectively, again comparing wild-type (A299) and A299S mutant models. This experimental design facilitated the disentanglement of lineage-specific background effects from the direct structural impact of the A/S substitution.

**FIG. 2.**
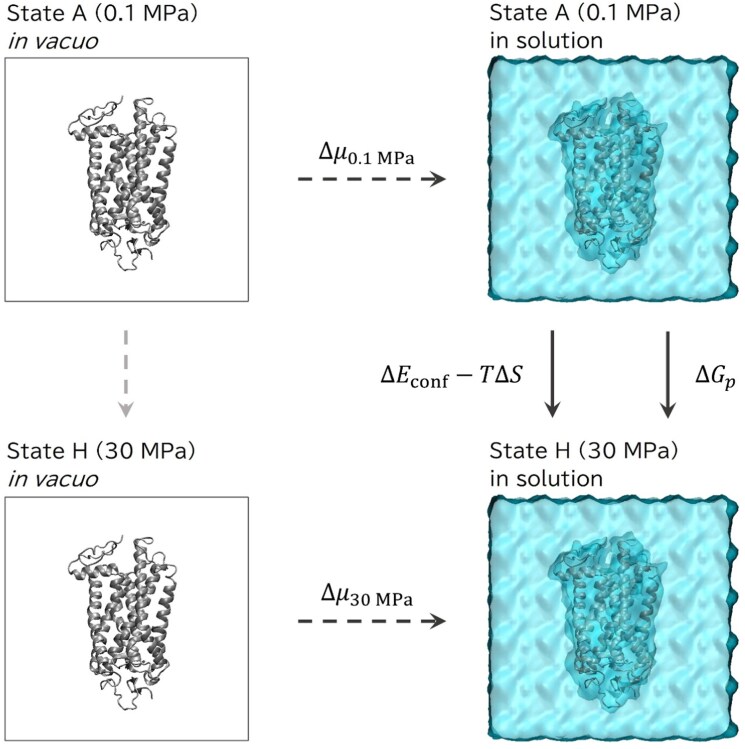
The thermodynamic cycle employed for the calculation of free energy shifts. Assuming the absence of structural alterations in rhodopsin throughout the solvation process, conformational energy and entropy in vacuo were determined using trajectories acquired in solution.

To capture the complementary dimensions of structural response, three classes of metrics derived from the simulation trajectories were analysed (see Materials and Methods). Firstly, isothermal compressibility (*κ_T_*) was calculated as a measure of global volume fluctuations. Secondly, residue-level flexibility was quantified through root-mean-square fluctuation (RMSF), allowing for an assessment of whether pressure alterations affected local dynamics. Lastly, pressure-induced free energy shifts (Δ*G_p_*) were estimated utilizing a thermodynamic cycle framework that decomposed stability modifications into conformational (Δ*E*_conf_), entropic (−*T*Δ*S*), and solvation free energy (ΔΔ*μ*) contributions (see Fig. 2). Collectively, this methodology offered a systematic means to evaluate the contribution of residue 299 to the pressure tolerance of rhodopsin.

**FIG. 3.**
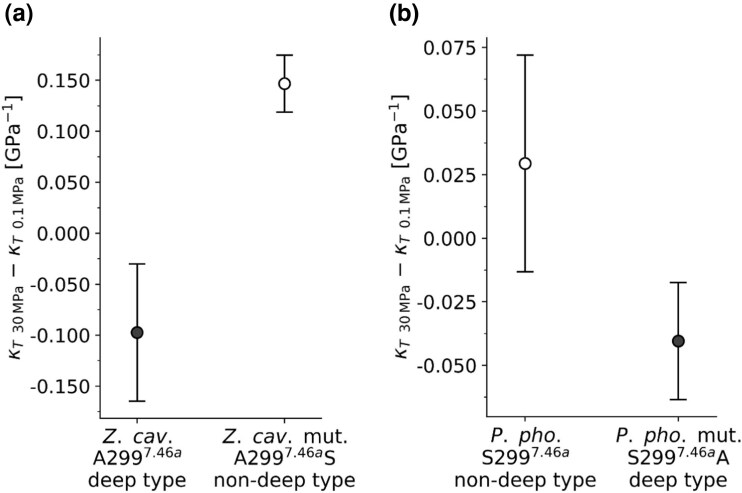
Differences in isothermal compressibility $\left(\kappa_{T}\right)$ between atmospheric (0.1 MPa) and high-pressure (30 MPa) conditions are shown for rhodopsins as the mean ± standard deviation (SD). (a) Data are presented for *Ziphius cavirostris* wild-type (A299, deep-diving type) and mutant (A299S, non-deep-diving type). (b) Data are depicted for *Phocoena phocoena* wild-type (S299, non-deep-diving type) and mutant (S299A, deep-diving type). The points represent the mean values, and vertical bars indicate the SD. The black-filled symbol denotes the deep-diving type (A299), and the open symbol denotes the non deep-diving type (S299).

The differences in *κ_T_* values between high pressure (30 MPa) and atmospheric pressure (0.1 MPa) showed consistent patterns across the rhodopsin variants (Fig. 3; Fig. S2; Table S5). In the deep-diving species *Z. cavirostris*, the wild-type rhodopsin containing A299 (−9.745 × 10^−2^ ± 6.727 × 10^−2^ GPa^−1^, mean ± SD) showed a smaller pressure-dependent change in *κ_T_* than the A299S mutant (1.467 × 10^−1^ ± 2.801 × 10^−2^ GPa^−1^), suggesting that introducing serine increased volume fluctuations under pressure (Fig. 3a; Table S5). Conversely, in the shallow-diving species *P. phocoena*, the reciprocal S299A mutation showed a smaller pressure-dependent change in *κ_T_* (−4.039 × 10^−2^ ± 2.303 × 10^−2^ GPa^−1^) than the wild type carrying S299 (2.945 × 10^−2^ ± 4.259 × 10^−2^ GPa^−1^), suggesting that the introduction of alanine reduced volume fluctuations under pressure (Fig. 3b; Table S5). Across the other rhodopsin models examined, we consistently found that variants retaining A299 exhibited smaller pressure-dependent changes in *κ_T_* than models retaining S299 (Fig. S2; Table S5). These consistent patterns demonstrate that residue 299 exerts an influence on the *κ_T_* of rhodopsin, with the presence of serine leading to greater compressibility under high pressure and alanine conferring reduced compressibility. Collectively, these findings suggest that the substitution at position 299 modulates global structural rigidity, aligning with its role in adapting to deep-sea pressure.

**FIG. 4.**
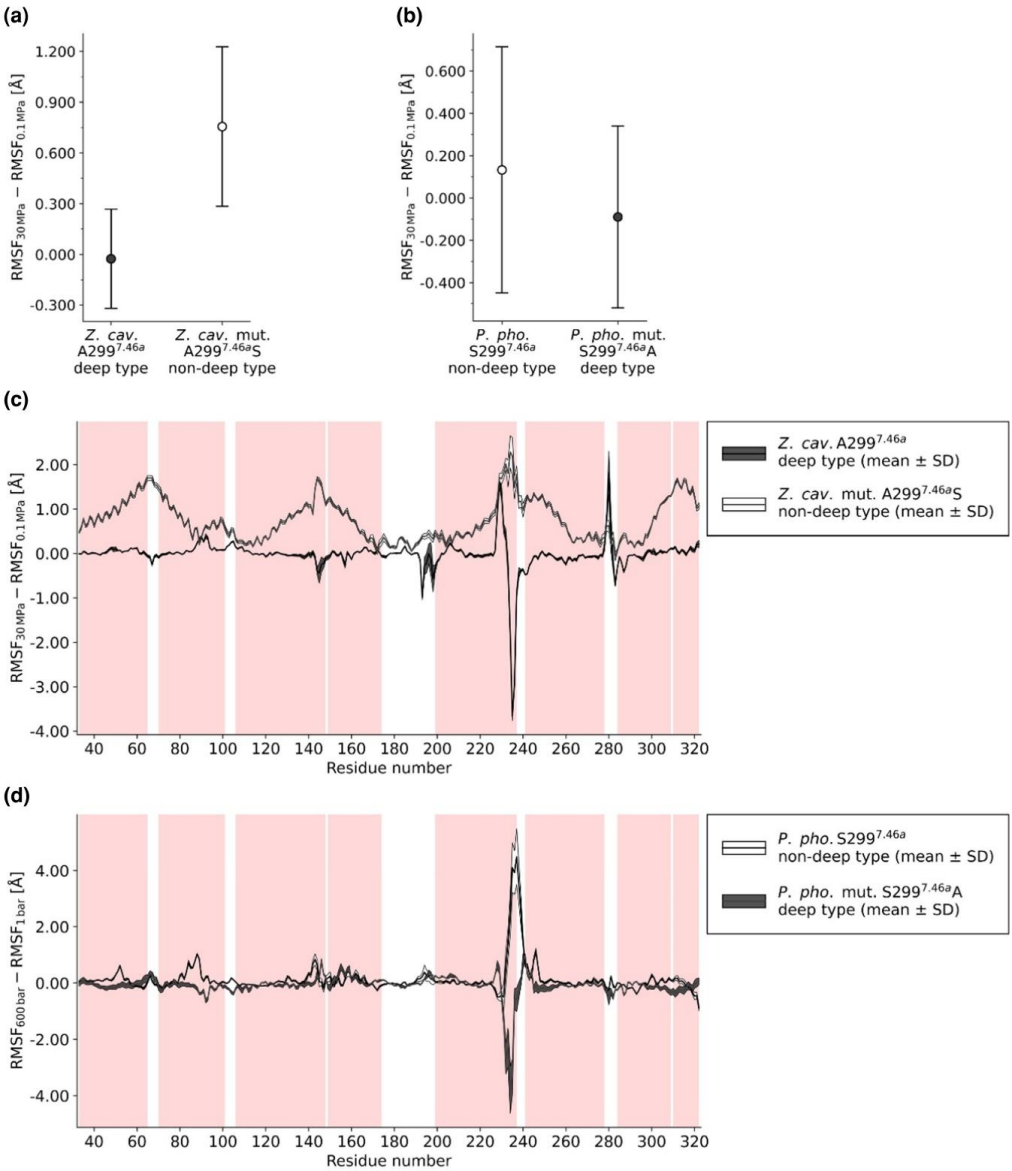
The effects of residue 299 on rhodopsin flexibility under high pressure. (a and b) Mean RMSF differences (RMSF_30 MPa_ − RMSF_0.1 MPa_) across helices I–VIII for wild-type and mutant rhodopsins as the mean ± standard deviation (SD). Symbols and error bars indicate means and SD. (c and d) Residue-level RMSF differences along helices I–VIII. Pink shading indicates helical regions. Black solid lines represent mean values, while the shaded areas indicate ± SD. (a and c) *Ziphius cavirostris*, and (b and d) *Phocoena phocoena*. The black symbols/shaded areas indicate deep-diving type (A299 in *Z. cavirostris* and S299A mutant in *P. phocoena*), and open symbols/shaded areas indicate non-deep-diving types (A299S mutant in *Z. cavirostris* and S299 in *P. phocoena*).

Residue-level flexibility was assessed as the pressure-dependent change in RMSF, calculated as ΔRMSF = RMSF_30 MPa_ − RMSF_0.1 MPa_. When averaged across helices I–VIII, ΔRMSF patterns were model-dependent, however, alanine at position 299 generally yielded smaller or slightly smaller pressure-dependent RMSF changes than serine (Fig. 4a and b; Fig. S3; Table S6). In *Z. cavirostris*, the A299 wild type showed a small ΔRMSF (−2.604 × 10^−2^ ± 2.939 × 10^−1^ Å), whereas the A299S mutant exhibited a substantially larger positive ΔRMSF (7.561 × 10^−1^ ± 4.711 × 10^−1^ Å), indicating a greater pressure-associated increase in flexibility upon serine introduction (Fig. 4a; Table S6). In *P. phocoena*, the difference between variants was less pronounced. The S299 wild type had a ΔRMSF of 1.329 × 10^−1^ ± 5.816 × 10^−1^ Å, and the reciprocal S299A mutant was slightly lower ΔRMSF (−8.985 × 10^−2^ ± 4.310 × 10^−1^ Å) (Fig. 4b; Table S6). Supplementary simulations of additional rhodopsin models further indicated that the magnitude of the effect varied across lineages. In particular, *L. vexillifer* and the reconstructed ancestral node leading to Ziphiidae showed a clear reduction in ΔRMSF for A299 models relative to their serine counterparts. On the other hand, *C. marginata* and the ancestral node leading to Physeteroidea exhibited only modest ΔRMSF reduction effects by alanin at this position (Fig. S3; Table S6). Taken together, while pressure-dependent RMSF across helices I–VIII responses were not uniformly distinct across all models, alanine at 299 consistently produced clearly or slightly smaller ΔRMSF values than serine at this position.

Analysis of RMSF variations across individual residues further demonstrated that this singular amino acid substitution exerted extensive influence on the structural dynamics (Fig. 4c and d; Fig. S4). The presence of alanine at position 299 was generally associated with reduced RMSF differences across multiple helical regions, whereas serine tended to augment flexibility under pressure (Fig. 4c and d; Fig. S4). Notably, these effects extended considerably beyond the immediate vicinity of residue 299, underscoring how a single amino acid modification can induce global changes in protein dynamics under high-pressure conditions. Despite the fact that residue 299 is located on helix VII, in *Z. cavirostris*, *P. phocoena*, and *C. marginata* the largest RMSF differences between the reciprocal A/S299 variants were observed in the region of helix V (Fig. 4c and d; Fig. S4). In the other models, no consistent spatial pattern on the rhodopsin structure was apparent for where RMSF differences were maximized, nevertheless, alanine at 299 generally produced smaller RMSF differences than the corresponding serine substitution across residues overall (Fig. S4). Collectively, these observations suggest that alanine at position 299 enhances pressure tolerance by mitigating pressure-induced increases in structural fluctuations, whereas serine undermines this capability.

**FIG. 5.**
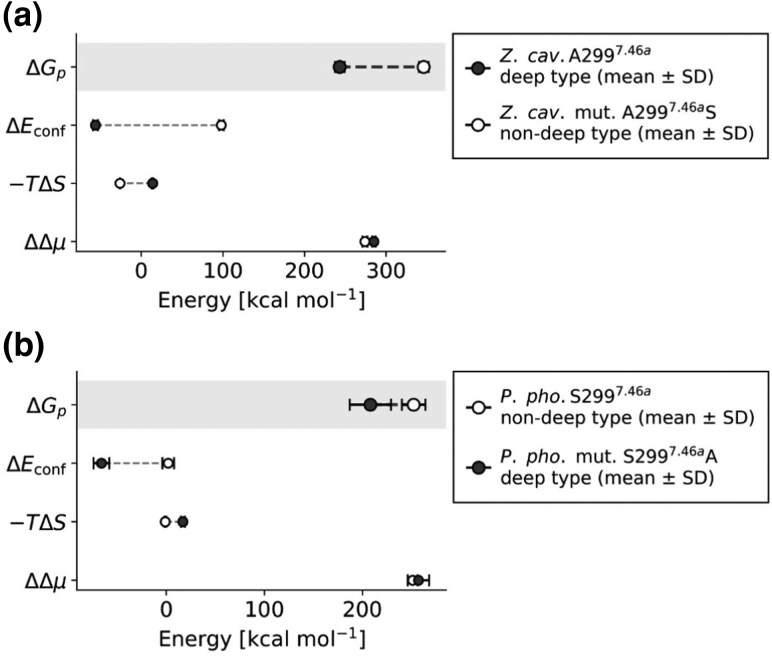
The pressure-dependent free-energy shift of A/S299 rhodopsin variants in (a) *Ziphius cavirostris* and (b) *Phocoena phocoena*. These dumbbell plots summarize the calculated free-energy shift components (mean ± SD, kcal mol^−1^) for wild-type and mutant rhodopsins. The top row in each panel highlights the overall pressure-dependent free-energy change (Δ*G_p_*). In contrast, the lower rows display the conformational energy shift (Δ*E*_conf_), the entropic shift term (−*T*Δ*S*), and the solvation free energy shift contribution (ΔΔ*μ*). Filled black circles indicate the deep-diving type (wild type A299 in *Z. cavirostris*, S299A mutant in *P. phocoena*), and open circles represent the non-deep-diving type (A299S in *Z. cavirostris* mutant and wild type S299 in *P. phocoena*, respectively).

To evaluate the energetic basis of pressure responses in deep-diving and non-deep-diving rhodopsins, we decomposed the calculated free-energy differences into conformational Δ*E*_conf_, entropic −*T*Δ*S*, and solvation free energy ΔΔ*μ* shift components (see materials and methods, Fig. 2). In the case of *Z. cavirostris*, the wild type (deep-diving type, A299) exhibited a substantially lower overall pressure-dependent free-energy change (Δ*G_p_* = 243 ± 6 kcal mol^−1^) compared to the non-deep-type mutant (A299S, 346 ± 6 kcal mol^−1^) (Fig. 5a; Table S7). This stabilizing effect was primarily driven by a favorable conformational energy shift (Δ*E*_conf_: deep-diving type, −56 ± 3 kcal mol^−1^; non-deep-diving type, 98 ± 3 kcal mol^−1^) (Fig. 5a; Table S7). The entropic contribution (−*T*Δ*S*: deep-diving type, 14 ± 1 kcal mol^−1^; non-deep-diving type, −26 ± 0 kcal mol^−1^) and solvation free energy shift term (ΔΔ*μ*: deep-diving type, 285 ± 2 kcal mol^−1^; non-deep-diving type, 274 ± 3 kcal mol^−1^) were comparatively minor (Fig. 5a; Table S7). The entropic term operated in opposing directions to conformational energy shift. In the deep-diving type, entropy decreased, thereby increasing the free energy, whereas in the non-deep-diving type, entropy increased and contributed to the reduction of the free energy (Fig. 5a; Table S7).

Conversely, in *P. phocoena*, the wild type (non-deep-diving variant, S299) demonstrated a larger Δ*G_p_* value (252 ± 12 kcal mol^−1^) compared to the mutant bearing the deep-diving residue (S299A, 208 ± 21 kcal mol^−1^) (Fig. 5b; Table S7). Therefore, in this shallow-diving species, the introduction of A299 again diminished the overall free-energy expense associated with pressurization, aligning with the pattern observed in *Z. cavirostris*. The energetic foundation of this reduction was comparable: advantageous alterations in conformational energy (Δ*E*_conf_: non-deep-diving type, 2 ± 6 kcal mol^−1^; deep-diving type, − 66 ± 8 kcal mol^−1^) explained most of the difference (Fig. 5b; Table S7). The entropic contribution (−*T*Δ*S*: non-deep-diving type, −1 ± 1 kcal mol^−1^; deep-diving type, 17 ± 2 kcal mol^−1^) was relatively minor yet differed in opposite directions between the two types (Fig. 5b; Table S7). Entropy decreased in the deep-diving variant, contributing to an increase in free energy shift, whereas it increased in the non-deep-diving variant, thereby partially counteracting the overall increase in free energy (Fig. 5b; Table S7). The solvation free energy shift (ΔΔ*μ*: non-deep-diving type, 251 ± 5 kcal mol^−1^; deep-diving type, 257 ± 11 kcal mol^−1^) had also relatively minor cotribution (Fig. 5b; Table S7).

Across the rhodopsin models supplementary examined, alanine at position 299 consistently reduced the overall pressure-dependent free-energy cost relative to the corresponding serine variant, indicating a broadly repeatable stabilizing effect of the alanin at this residue (Fig. S5; Table S7). In all models, this stabilization was driven primarily by a more favorable Δ*E*_conf_, mirroring the pattern observed in *Z. cavirostris* and *P. phocoena* (Fig. S5; Table S7). By contrast, the contribution of the ΔΔ*μ* was more variable among models. In *L. vexillifer* and in the reconstructed ancestral rhodopsins for the nodes leading to Physeteroidea and Ziphiidae, A299 was associated with a more favorable ΔΔμ component than S299, contributing additional stabilization under pressure (Fig. S5; Table S7). Conversely, in *C. marginata*, the ΔΔμ term favored the A299S mutant over the A299 wild type (Fig. S5; Table S7). Taken together, evaluation of the pressure-dependent free-energy shift supports the conclusion that alanine at 299 confers enhanced pressure tolerance relative to serine at this position across the tested models, primarily by stabilizing the conformational energy component under high-pressure conditions. This consistent contrast between the A299 and S299 states highlights the A/S substitution at residue 299 as a key determinant of rhodopsin performance under high hydrostatic pressure.

### Discussion

This study provides molecular evidence that residue 299^7.46a^ of rhodopsin constitutes a critical determinant of pressure tolerance in deep-diving cetaceans. By integrating codon substitution models with molecular dynamics simulations, it was consistently observed that the presence of alanine at this site (A299) enhances structural robustness against elevated hydrostatic pressure, while serine (S299) increases conformational flexibility and amplifies pressure-induced destabilization. Importantly, this pattern was evident in *Z. cavirostris* and *P. phocoena*, and supplementary analyses of additional extant and reconstructed ancestral rhodopsin models yielded broadly consistent results, strengthening support for a mechanism centered on the A/S299 substitution.

Our codon-based evolutionary analysis revealed that amino acid substitutions linked to substantial changes in amino acid compressibility (*K^0^*) accrued at notably higher rates along the deep-diving lineages of Physeteroidea and Ziphiidae in comparison to background lineages. This observation implies that, during the process of adaptation to extreme hydrostatic pressures, substitutions affecting *K^0^* were preferentially preserved, signifying that structural attributes associated with pressure tolerance may have been a significant focus of selection in deep-diving cetaceans.

Methodologically, our analysis emphasizes the significance of the physicochemical property-based codon substitution model proposed by Wong, Sainudiin, and Nielsen (2006). Unlike the conventional Goldman and Yang (1994) model, which deduces positive selection solely from increased nonsynonymous to synonymous substitution rates, the WSN06 framework expands this concept to include amino acid physicochemical properties. By categorizing nonsynonymous substitutions into conservative or radical modifications relative to a user-defined trait, the model facilitates the interpretation of selection through a tangible structural property—in this instance, *K^0^*, which may be associated with hydrostatic pressure tolerance. Consequently, property-based models serve as a vital methodological link between molecular evolution and structural biology.

At the same time, it is essential to acknowledge several caveats. As Wong et al. (2006) advised, the model cannot determine solely from the data which property constitutes the true target of selection, given that amino acids differing in one property often vary in others. Consequently, the selection signals attributed here to *K^0^* may also be indicative of correlated traits, and the observed *γ* values should not be construed as definitive evidence that *K^0^* is the sole target of selection. Furthermore, *K^0^* does not correspond in a straightforward one-to-one fashion to whole-protein compressibility. Empirical prediction models for protein adiabatic compressibility have demonstrated that multiple physicochemical features, rather than *K^0^* alone, are required for accurate estimation (Gromiha and Ponnuswamy 1993). Thus, the functional significance of substitutions associated with compressibility must be validated through actual physical measurements, such as molecular dynamics simulations, as conducted in this study.

Within this framework, our analyses identified residue 299 as a particularly strong candidate for adaptation to hydrostatic pressure. Notably, the S299A substitution was independently detected in both Physeteroidea and Ziphiidae. Beyond cetaceans, the same S299A substitution has been documented in deep-sea fishes (Varela and Ritchie 2015) and in deep-diving pinnipeds, such as elephant seals (Xia et al. 2021), indicating that this site may represent a recurrent target of deep-sea adaptation across distantly related vertebrates. In cetaceans specifically, residue 299 predominantly occurs as either serine or alanine, with serine being inferred as the ancestral state (Dungan and Chang 2017). Due to the limited extant odontocete lineages, with the genus *Platanista* being the sole lineage positioned between Physeteroidea and Ziphiidae, multiple evolutionary scenarios remain plausible concerning the occurrence of A299. The pattern observed could reflect genuine convergence in the two deep-diving clades or an independent A299S substitution event within the *Platanista* lineage and the last common ancestor of the families Iniidae, Delphinidae, Monodontidae, and Phocoenidae. However, given the current taxonomic sampling, distinguishing among these possibilities remains a challenge.

Our molecular dynamics analyses consistently demonstrated that rhodopsins containing alanine at position 299 exhibited diminished sensitivity to hydrostatic pressure in comparison with serine-bearing variants. Specifically, A299 rhodopsins displayed lower isothermal compressibility (*κ_T_*), decreased residue-level fluctuations (RMSF), and reduced entropy under high pressure relative to atmospheric conditions. Furthermore, the pressure-induced increase in free energy (Δ*G_p_*) was markedly smaller in A299 variants than in their S299 counterparts. Therefore, the substitution of alanine appears to confer stability to rhodopsin by dampening pressure-driven structural fluctuations and minimizing the energetic penalty associated with compression.

The interpretation of our results can be situated within a thermodynamic framework. According to Le Chatelier's principle, a system at equilibrium subjected to increased pressure will shift toward states associated with volume reduction, formally expressed as (∂Δ*G*/∂*p*)_T_ = Δ*V*, where Δ*G* represents the Gibbs free energy, and Δ*V* is the volume change from reactants to products, *P* indicates pressure, and *T* signifies temperature. In the context of protein folding, most proteins exhibit a negative Δ*V* upon unfolding (Chalikian 2003), indicating that the unfolded conformation occupies a smaller volume than the native state; consequently, increased pressure promotes unfolding. One hypothesis for adaptation to high-pressure environments posits that proteins in deep-sea organisms have evolved to undergo smaller negative or even positive volume changes during the folding process. However, comparative proteomic analyses of piezophilic and non-piezophilic bacteria and archaea have reported minimal differences in overall Δ*V* values (Avagyan et al. 2020). Instead, case studies of individual proteins suggest that pressure tolerance often results from modifications in compressibility or energetic stability rather than simple volume effects. For instance, lactate dehydrogenases of the deep-sea fish *Coryphaenoides armatus* resist pressure-induced denaturation not by reducing Δ*V* but through variations in compressibility, as highlighted by Maguire et al. (2024). Similarly, α-actin monomers from *C. armatus* and *C. yaquinae* exhibited no significant differences in volume or compressibility compared to shallow-water relatives; nevertheless, they displayed more favorable pressure-dependent shifts in free energy, with conformational energy (Δ*E*_conf_) serving as the primary contributing factor (Wakai et al. 2014).

In accordance with established precedents, our results suggest that cetacean rhodopsins can acquire robustness to pressurization through modifications in compressibility and energetic stability. Importantly, cetaceans do not inhabit a single, constant high-pressure environment, instead, they repeatedly experience large pressure changes during dives while still requiring rhodopsin function near the sea surface. Thus, we interpret “pressure tolerance” in an explicitly differential sense, Δ*G_p_* quantified the energetic penalty of increasing pressure (from 0.1 to 30 MPa). Under this definition, the smaller Δ*G_p_* observed for the deep-diving type (A299) relative to the non-deep-diving type (S299) indicates reduced pressure sensitivity, rather than specialization to extreme depths alone. This stabilizing effect was predominantly driven by a favorable conformational energy shift (Δ*E*_conf_), resembling the energetic mechanism proposed for α-actin in deep-sea fish (Wakai et al. 2014). Our results support the conclusion that the A299 mitigates pressure-induced structural destabilization, a property that could be advantageous for proteins operating across repeatedly changing hydrostatic pressures during diving.

Nonetheless, several limitations of our *in silico* approach warrant consideration. Firstly, our analyses are solely based on computational simulations rather than direct experimental data. Secondly, due to technical constraints in free energy calculations, rhodopsins were modeled in aqueous solution instead of within lipid bilayers, despite being integral membrane proteins. Thirdly, the chromophore retinal, which forms a Schiff base bond with K296^7.42a^ and is vital for light absorption, was omitted. Lastly, we modeled rhodopsins only in their dark state using bovine templates, leaving unresolved the potential impact of hydrostatic pressure on conformational changes during photoactivation. These limitations serve as a caution against over-interpretation, although they do not undermine the consistent patterns of pressure resilience observed in our analyses. These limitations are likely to have a disproportionate impact on residue-level flexibility metrics (RMSF) and solvation-related components (ΔΔ*μ*). Accordingly, the model-dependent and sometimes non-uniform trends observed for RMSF and ΔΔ*μ* should be interpreted cautiously and primarily in a qualitative sense. In addition, the 50 ns trajectories analysed here are unlikely to exhaustively sample the conformational ensemble relevant to rhodopsin dynamics—particularly the large-scale transitions associated with photoactivation in a native lipid bilayer. Future work combining membrane-embedded and retinal-bound models, with longer and replicated simulations and enhanced-sampling approaches such as guided sampling based on essential dynamics (e.g. Linder et al. 2013), could more efficiently characterize conformational transition pathways and the underlying energy landscapes of the A/S299 variants under pressure.

Previous research on cetacean rhodopsins has primarily concentrated on spectral tuning in relation to underwater light environments. Substitutions at residues 83^2.50a^ and 292^7.38a^ significantly account for much of the interspecific variation observed in absorption maxima, whereas residue 299 was regarded as having limited influence on spectral characteristics (Dungan et al. 2016). Conversely, functional assays conducted on killer whale rhodopsin demonstrated that the S299A mutation can interact epistatically with residue 83^2.50a^ to alter retinal release kinetics, thereby potentially affecting dark adaptation (Dungan and Chang 2017). Importantly, these effects need not be mutually exclusive: selection at residue 299 may reflect combined pressures acting on rhodopsin performance, including visual ecology, dark-adaptation kinetics, and hydrostatic-pressure resilience, with possible epistatic contingencies. Notably, the occurrence of the S299A substitution in deep-sea fishes that likely experience chronic darkness (Varela and Ritchie 2015) suggests that an explanation based solely on dark-adaptation kinetics may be incomplete, and is consistent with the possibility that pressure-related constraints can also favor A299. While it remains difficult to distinguish whether the modest differences among cetacean lineages in our simulations reflect true lineage-specific effects (background sequence) or incomplete sampling of conformational ensemble, the overall pattern supports the interpretation that residue 299 contributes to rhodopsin adaptation through mechanisms that extend beyond spectral tuning and may include enhanced stability under pressure.

The occurrence of A299 in non-deep-diving taxa does not necessarily contradict a role in pressure robustness. From a mutational perspective, serine and alanine can be interconverted by a single nucleotide substitution, making A/S299 a readily accessible variant in many lineages. Accordingly, A299 is also present in some terrestrial mammals (e.g. bovine rhodopsin). In such contexts, A299 could reflect neutral drift or selection acting on other aspects of rhodopsin function, rather than hydrostatic pressure per se. However, in species that routinely experience elevated hydrostatic pressure during deep dives, even modest advantages in pressure robustness could bias retention of A299 over S299. Thus, the presence of A299 across a broad taxonomic range is compatible with an explanation in which this substitution is easy to arise, but is more likely to be maintained by selection in ecological settings where pressure-related constraints are recurrent and physiologically relevant.

Interpreting the reconstructed ancestral rhodopsins in an explicit ecological framework is challenging because ancestral diving traits for the focal nodes cannot be reliably inferred at present. This limitation is particularly acute for Physeteroidea, whose evolutionary history is complex and incompletely resolved: the group comprises a paraphyletic stem physeteroids, crown Physeteridae, and the monophyletic Kogiidae (Paolucci et al 2020). Many physeteroid fossils, including extinct members of crown Physeteridae and Kogiidae, have been recovered from shallow-marine deposits such as those of the Pisco Basin (e.g. Paolucci et al 2020). Given this incomplete and potentially biased record, discussion of the diving ecology of the lineage ancestral to extant physeteroids remains limited. For Ziphiidae, the fossil record more often places many crown ziphiids in deep-sea depositional settings, which is consistent with the possibility that the common ancestor of crown ziphiids had already evolved deep-diving traits (e.g. Lambert et al. 2015). At the same time, evidence of shallow-water foraging exists for some stem ziphiids, and a prevailing view is that pronounced deep-diving adaptation characterizes primarily crown ziphiids (Lambert et al. 2015). Therefore, while the ancestral sequence we modeled for Ziphiidae may plausibly approximate the stage near the origin of crown ziphiids—and thus could correspond to a deep-diving ecological regime—this connection remains inferential. Moreover, because our analyses indicate that A299 tends to confer a favorable pressure response across all tested backgrounds (including extant non–deep-diving taxa and reconstructed ancestors), it is difficult to directly map the rhodopsin pressure-response signature onto a specific ancestral diving phenotype within this framework. In this sense, the ancestral reconstructions strengthen the generality of the A/S299 effect across divergent sequence backgrounds, while the fossil and trait uncertainties caution against over-interpreting these ancestral models as direct proxies for the depth ecology of particular ancestral lineages.

Our findings offer a novel perspective, extending beyond the roles in spectral tuning or dark adaptation, as the S/A299 polymorphism may directly contribute to hydrostatic pressure tolerance. To our knowledge, this constitutes the first evidence that the S299A mutation has influenced the structural robustness of cetacean rhodopsins under hydrostatic pressure. The idea that pressure can affect rhodopsin dynamics is further corroborated by experimental studies in other systems, where extremely high pressures alter conformational intermediates during photoactivation and even absorption spectra in squid rhodopsins (r-opsin) and bacteriorhodopsins (Tsuda et al. 1977; Tsuda and Ebrey 1980; Kovacs et al. 1993). Although these effects were observed under hundreds of megapascals and different opsin clades, they demonstrate that rhodopsins are inherently pressure-sensitive proteins.

In summary, our findings indicate that the recurrent S299A substitution in deep-diving cetaceans may enhance the pressure tolerance of rhodopsin, thereby providing structural stability in environments where both light availability and hydrostatic pressure impose significant constraints. This insight provides a novel perspective on the evolutionary history of cetacean vision; aside from spectral adaptation to the light field, rhodopsins may also have undergone structural modifications, enabling their functionality under extreme hydrostatic pressure conditions. More broadly, our comprehensive approach—integrating physicochemical property-based evolutionary models with molecular dynamics simulations—serves as a framework for elucidating how natural selection influences protein stability in extreme environments. Future applications of this methodology to other proteins may uncover overarching principles of vertebrate adaptation to the deep-sea environment.

## Materials and Methods

### Rhodopsin Sequence Collection

The coding sequence (CDS) of *Tursiops truncatus* rhodopsin (accession No: NM_001280659.1) was utilized as a query nucleotide sequence. Initially, candidate genomic regions encompassing intronic and exonic sequences were identified from the genomes of the 51 cetacean species and *Hippopotamus amphibius*, one of the most closely related extant species, through the execution of the BLASTN program (nucleotide-nucleotide BLAST v2.15.0+) (Altschul et al. 1990; Zhang et al. 2000; Camacho et al. 2009; Zhou et al. 2013; Keane et al. 2015; Kishida et al. 2015; Dudchenko et al. 2017; Jones et al. 2017; Autenrieth et al. 2018; Dudchenko et al. 2018; Vijay et al. 2018; Fan et al. 2019; Ming et al. 2019; Westbury et al. 2019; Zoonomia Consortium 2020; Morin et al. 2021; Yuan et al. 2021; Foote et al. 2022; Wolf et al. 2022, 2023; Yin et al. 2022; Brownlow et al. 2024; Bukhman et al. 2024; Carminati et al. 2024; Ding et al. 2024; Feyrer et al. 2024; Davison et al. 2024a, 2024b; Lin et al. 2025; Sharma et al. 2025). The list of genome assemblies employed in this study is presented in Table S1.

For *Platanista minor*, which possesses a low-coverage genome assembly (28x), the BLASTN search against genome contigs did not successfully retrieve the complete CDS, as the exonic regions were dispersed across multiple contigs rather than being contained within a single contig. Consequently, a BLASTN search was conducted directly against the whole genome shotgun data (SRX8010159) via the NCBI web BLAST interface. Gene regions that spanned fragmented contigs were manually reconstructed by aligning and merging the BLAST hits obtained from the contigs.

When genomic regions of candidates were obtained in reverse orientation or as complements, an in-house C++ script was utilized to convert the sequences to the correct strand orientation. Gene structure prediction was subsequently conducted using the FATE v2.8.0 program, with genewise (wise2-4-1) specified as the underlying algorithm, to determine intron-exon boundaries within the retrieved regions (Birney et al. 2004; Camacho et al. 2009; Moriya-Ito et al. 2018). Ultimately, based on the predicted gene structures, the CDSs were extracted employing the getfasta command in bedtools v2.31.1 (Quinlan 2014). To verify that the retrieved sequences represented rhodopsin rather than other genes, such as cone opsins or non-visual opsins, a reciprocal BLAST search was performed; each CDS retrieved was used as a query against the *T. truncatus* genome, and the sequence was classified as a rhodopsin if the top hit corresponded to rhodopsin.

### Evolutionary Analysis of Amino Acid Compressibility Using Codon Substitution Models

The rate of amino acid substitution in genes is well established to depend on the physicochemical properties of amino acids, which can directly influence protein structure and function (e.g. Epstein 1967; Clarke 1970). In this study, our objective was to identify selection acting upon a specific physicochemical property within rhodopsin using a maximum likelihood framework.

As the physicochemical property of interest, we focused on amino acid compressibility (*K^0^*), which is defined as the relative increase in the volume of the system per unit decrease in pressure (m^3^ mol^−1^ Pa^−1^ × 10^−15^) (Gromiha and Ponnuswamy 1993). It is hypothesized that in deep-diving cetaceans, amino acid substitutions tend to favor residues with lower absolute compressibility changes, indicative of reduced structural deformation under pressure.

To evaluate this hypothesis, we utilized the model proposed by Wong, Sainudiin, and Nielsen (2006). This model classifies nonsynonymous codon substitutions into two types: conservative substitutions, which maintain the targeted amino acid property, and radical substitutions, which induce a significant alteration in the property. Specifically, the model allows for three types of substitutions: synonymous substitutions, conservative nonsynonymous substitutions, and radical nonsynonymous substitutions. The rates of conservative and radical nonsynonymous substitutions are scaled relative to the synonymous substitution rate, with parameters *ω* and *γ*, respectively. Nonsynonymous codon substitutions were classified according to the magnitude of change in *K^0^*. We generated a list of nonsynonymous substitutions accessible via a single nucleotide change and computed the absolute difference in *K^0^* (|Δ*K^0^*|) for each substitution. Because defining an a priori biologically grounded partition between radical and conservative changes is non-trivial, we evaluated a series of nine candidate partitions in which the top *P*% of substitutions ranked by |Δ*K^0^*| were designated as radical (with *P* = 10, 20, …, 80, 90), and the remaining substitutions were classified as conservative. We then tested the partition that provided the best model fit based on likelihood comparisons.

The baseline codon substitution model specifies the instantaneous substitution rate from sense codon *i* to sense codon *j* as


(1) $$\begin{array}{r} q_{i,j}=\{\begin{array}{cc} 0 & \mathrm{if}\;i\;\mathrm{and}\;j\;\mathrm{differ}\;\mathrm{at}\;\mathrm{more}\;\mathrm{than}\;\mathrm{one}\;\mathrm{position},\\ \pi_{j} & \mathrm{for}\;\mathrm{synonymous}\;\mathrm{transversion},\\ \kappa \pi_{j} & \mathrm{for}\;\mathrm{synonymous}\;\mathrm{transition},\\ \gamma_{ij}\omega_{ij}\pi_{j} & \mathrm{for}\;\mathrm{non}\text{-}\mathrm{synonymous}\;\mathrm{transversion},\\ \gamma_{ij}\omega_{ij}\kappa \pi_{j} & \mathrm{for}\;\mathrm{non}\text{-}\mathrm{synonymous}\;\mathrm{transition}, \end{array} \end{array}$$

$$\mathrm{where}\{\begin{array}{c} \gamma_{ij}=1\;\mathrm{and}\;\omega_{ij}=\omega ,\;\mathrm{if}\;i\;\mathrm{and}\;j\;\mathrm{are}\;\mathrm{conservative}\;\mathrm{substitution},\\ \gamma_{ij}=\gamma \;\mathrm{and}\;\omega_{ij}=1,\;\mathrm{if}\;i\;\mathrm{and}\;j\;\mathrm{are}\;\mathrm{radical}\;\mathrm{substitution}. \end{array}$$

where *κ* is the transition-transversion rate ratio, and *π_j_* is the equilibrium frequency of codon *j* (Wong et al. 2006). Under the constraint *γ* = *ω*, the WSN06 model reduces to the standard Goldman and Yang (1994) codon model (Goldman and Yang 1994).

Both the branch and site models were employed to examine signatures of selection. For the branch model analysis, branches leading to Physeteroidea and Ziphiidae were designated as foreground (fg) branches. In contrast, all other branches served as background (bg) branches (Fig. 1). An alternative model permitting the separate estimation of *γ*_fg_ and *γ*_bg_ was compared to a null model assuming a common *γ* across all branches, using AIC comparison and LRT with a chi-square approximation. According to our hypothesis, the estimated *γ*_fg_ values for the branches leading to Physeteroidea and Ziphiidae were anticipated to exceed the *γ*_bg_ values assigned to the background branches. This outcome would suggest that substitutions inducing radical changes in compressibility were selectively favored along the lineages of deep-diving cetaceans.

In the site model analysis, we allowed *ω* and *γ* to vary between two classes: *ω_0_* ≤ 1 or *ω*_1_ > 1, as well as *γ*_0_ ≤ 1 or *γ*_1_ > 1. Thus, there were four possible site classes, combining different *ω* and *γ* categories.


(2) $$\begin{array}{c} \{\begin{array}{c} (i)\omega_{0}\leq 1,\;\gamma_{0}\leq 1,\\ (\mathrm{ii})\omega_{0}\leq 1,\;\gamma_{1}>1,\\ (\mathrm{iii})\omega_{1}>1,\;\gamma_{0}\leq 1,\\ (\mathrm{iv})\omega_{1}>1,\;\gamma_{1}>1. \end{array} \end{array}$$

The proportions of each category and the posterior probabilities of each site belonging to each site class were obtained. The AIC comparison and LRT examined whether selection preferentially favored radical substitutions, i.e. those inducing significant changes in compressibility, by comparing a null mixture model containing only site classes with *γ* ≤ 1 (specifically, site classes (i) and (iii)) to an alternative model that permits all four site classes.

For parameter estimation, the initial phylogenetic tree was reconstructed using IQ-TREE3 v3.0.1, based on the Goldman and Yang codon model (Goldman and Yang 1994; Minh et al. 2020; Wong et al. 2025). The input topology for the phylogenetic tree was assembled using the TimeTree of Life resource (Kumar et al. 2022). The phylogenetic placement of Rice's whale (*Balaenoptera ricei*; Rosel et al. 2021), which was described as a new species in 2021, was assigned based on the whole-genome phylogeny reported by Wolf et al. (2023). Parameter optimization was conducted using a modified version of the EvoRadical program (Wong et al. 2006), in which a branch model implementation was incorporated in this study, as the original software supported only site models.

Furthermore, ancestral sequences for the nodes leading to Physeteroidea and Ziphiidae were reconstructed utilizing IQ-TREE3 v3.0.1 under the best-fitting TN + F + I + G4 substitution model selected by ModelFinder implemented in IQ-TREE3 (Tamura and Nei 1993; Yang 1994; Kalyaanamoorthy et al. 2017; Minh et al. 2020; Wong et al. 2025; Fig. 1). Based on these reconstructions, the ancestral states of *K^0^* at each codon site were inferred. Sites exhibiting convergent amino acid changes independently in Physeteroidea and Ziphiidae were identified. Among these sites, we further screened for codon sites assigned with a posterior probability >95% to site classes associated with *γ*_1_ > 1 (i.e. site classes (ii) and (iv)) under the site model. From this subset, amino acid substitutions that diminished the *K^0^* value were designated as candidate mutations potentially conferring structural robustness against hydrostatic pressure in deep-diving cetaceans. Interactive Tree Of Life (iTOL) was used to visualize the phylogenetic trees (Letunic and Bork 2024).

### Molecular Dynamics Simulations

Molecular dynamics simulations were conducted to examine the structural robustness and free energy change of rhodopsin under high-pressure conditions in comparison to those at atmospheric pressure. Rhodopsin structures from Cuvier's beaked whale (*Z. cavirostris*), representative of a deep-sea species, and harbor porpoise (*P. phocoena*), indicative of a non-deep-diving species, were analysed. Additionally, we developed structural models incorporating specific mutations at candidate sites identified through codon-based evolutionary analyses as potentially contributing to hydrostatic pressure tolerance. In *Z. cavirostris*, the A299S mutation was introduced to potentially diminish structural robustness and modify free energy change under high pressure. Conversely, in *P. phocoena*, the S299A mutation, potentially enhancing pressure tolerance, was introduced.

To assess the robustness of these patterns, we performed supplementary analyses on additional taxa and reconstructed ancestral proteins. Specifically, we analysed two non–deep-diving cetaceans that nonetheless retain A299—baiji (*L. vexillifer*) and pygmy right whale (*C. marginata*)—by comparing the wild-type (A299) structures with corresponding A299S mutant models. We further analysed reconstructed ancestral rhodopsins for the nodes leading to the deep-diving lineages Physeteroidea and Ziphiidae, respectively, again comparing the inferred wild-type (A299) ancestral structures with A299S mutants. Ancestral sequences were reconstructed in IQ-TREE3 v3.0.1 based on the most probable nucleotide at each site, under the best-fitting TN + F + I + G4 substitution model selected by ModelFinder.

Three-dimensional structural models of rhodopsin were constructed utilizing the crystal structure of dark-state bovine rhodopsin (PDB ID: 1U19) as the modeling template (Okada et al. 2004), following the standard modeling procedure with the SWISS-MODEL web server (https://swissmodel.expasy.org/) (Waterhouse et al. 2018). The protonation states of rhodopsin residues at pH 7.4 were assessed using the CHARMM-GUI Solution Builder (Jo et al. 2008), and residue E122 was substituted with its protonated form (GLUP). The orientation of the rhodopsin model was subsequently refined using the PPM 2.0 web server (https://opm.phar.umich.edu/ppm_server) (Lomize et al. 2012).

Molecular dynamics simulations of the rhodopsin models were conducted utilizing GROMACS v2025.1 (Abraham 2015). SPC/E water served as the solvent (Berendsen et al. 1987), and the CHARMM36m force field was applied to all molecular components (Huang et al. 2017). The system was positioned within a simulated cubic box, maintaining a minimum distance of 10 Å between the protein and the box boundaries. It was solvated with water enhanced with 0.15 M NaCl. Each structural model underwent simulation under two pressure conditions: atmospheric pressure (0.1 MPa) and elevated pressure (30 MPa). Simulations were conducted with a time step of 2 fs.

Prior to initiating production runs, each system underwent standard energy minimization using the steepest descent algorithm until the maximum force was reduced below 1,000 kJ mol^−1^ nm^−1^. Subsequently, a 200 ps equilibration was conducted under the NVT ensemble at 300 K employing the velocity-rescale thermostat (Bussi et al. 2007). This was followed by an NPT equilibration under isotropic pressure coupling using the C-rescale barostat at 0.1 MPa for 200 ps (Bernetti and Bussi 2020). For simulations conducted at 30 MPa, the pressure was gradually increased following the initial 0.1 MPa equilibration. The pressure was raised in 0.5 MPa increments every 30 ps until reaching 30 MPa, thereby preventing structural artifacts due to abrupt pressurization. The final NPT equilibration was performed at 30 MPa for 200 ps. Electrostatic interactions were calculated utilizing the Particle Mesh Ewald (PME) method, with a real-space cutoff of 10 Å (Darden et al. 1993; York et al. 1993). Van der Waals interactions were managed with a force-switch method, truncated at 10 Å (Steinbach and Brooks 1994). For each system, a production run of 100 ns was conducted at pressures of 0.1 and 30 MPa, employing isotropic pressure coupling via the Parrinello–Rahman barostat (Parrinello and Rahman 1980). Coordinate and energy data were saved every 2 ps. Analysis was performed on the final 50 ns of each trajectory (50–100 ns) to mitigate the effects of initial equilibration. Structural visualization was carried out using VMD v1.9.4a55 (Humphrey et al. 1996).

To assess the structural response of rhodopsin to elevated pressure conditions, we performed three complementary analyses on the molecular dynamics trajectories. Initially, we computed the isothermal compressibility (*κ_T_*) as an indicator of overall volume fluctuations. Subsequently, we assessed residue-level flexibility by comparing RMSF values between 0.1 and 30 MPa. Finally, as part of a comprehensive thermodynamic evaluation, we estimated free energy differences by decomposing contributions from conformational energy, entropy, and solvation free energy between simulations at 0.1 and 30 MPa.

Isothermal compressibility (*κ_T_*) was calculated according to the following definition:


(3) $$\begin{array}{c} \kappa_{T}=-\frac{1}{V}{\left(\frac{\partial V}{\partial p}\right)}_{T}=\frac{\langle V^{2}\rangle -\langle V\rangle^{2}}{k_{B}T\langle V\rangle } \end{array}$$

where *V*, *p*, *k_B,_* and *T* denote the solute volume, system pressure, Boltzmann constant, and absolute system temperature, respectively. Angle brackets indicate ensemble averages, which were calculated in this study over the final 50 ns of each simulation trajectory. *V*, *p,* and *T* data were obtained using standard analysis gmx command tools implemented in GROMACS. The differences of isothermal compressibility, defined as (*κ_T_* in 30 MPa condition − *κ_T_* in 0.1 MPa condition) were subsequently calculated.

RMSF of Cα values were calculated using the gmx rmsf utility within GROMACS. To address the limited reliability of the predicted three-dimensional structures in the N- and C-terminal regions of rhodopsin, only residues from E33^1.28a^ to C322^8.59a^, corresponding to helices I–VIII, were incorporated in the analysis. RMSF values were compared for each residue as well as for the average across the analysed region, at pressures of 0.1 and 30 MPa. The differences, defined as (RMSF in 30 MPa condition − RMSF in 0.1 MPa condition) were subsequently calculated.

In accordance with the overarching framework established by Wakai et al. (2014), the shift in free energy associated with the increase in pressure from 0.1 MPa (state A) to 30 MPa (state H), hereinafter designated as Δ*G_p_*, was estimated using a thermodynamic cycle as illustrated in Fig. 2. Within this scheme, state A and state H denote the equilibrium conformations of rhodopsin under atmospheric and high-pressure conditions, respectively. It is assumed that the solvation process does not modify the protein structure. Consequently, the cycle yields the following relation,


(4) $$\begin{array}{rl} \Delta G_{p} & =\Delta E_{\mathrm{conf}}-T\Delta S+(\Delta \mu_{30\;\mathrm{MPa}}-\Delta \mu_{0.1\;\mathrm{MPa}})\\ & =\Delta E_{\mathrm{conf}}-T\Delta S+\Delta \Delta \mu \end{array}$$

where Δ*E*_conf_ is the change in solute conformational energy, Δ*S* is the solute entropy difference, and ΔΔ*μ* is the change in solvation free energy between 0.1 and 30 MPa.

Δ*E*_conf_ was derived from the averages of the conformational energy of the solute, computed utilizing the gmx energy utility applied to molecular dynamics trajectories. The conformational energy was calculated as the sum of bonded interactions (including bond stretching, Urey–Bradley, proper dihedral, improper dihedral, and CMAP dihedral) and intramolecular nonbonded interactions (comprising short-range Coulomb and Lennard–Jones interactions, as well as 1–4 Coulomb and Lennard–Jones interactions). Within the thermodynamic framework of this study, solvation processes are defined to preserve the structural integrity of rhodopsin. Accordingly, Δ*E*_conf_ in vacuo was determined using conformations obtained from MD simulations conducted in an aqueous environment.

The solvation free energy (Δ*μ*) was partitioned into polar and nonpolar components following the methodology outlined in Lee and Olson (2013). The polar component was computed by solving the Poisson–Boltzmann equation utilizing the APBS software package (Holst and Saied 1993; Bank and Holst 2003; Jurrus et al. 2018). The dielectric constants for water at pressures of 0.1 and 30 MPa were set to 77.75 and 78.83, respectively, in accordance with the reports of Fernandez et al. (1997). The nonpolar contribution can be approximately subdivided into three components (Wagoner and Baker 2006),


(5) $$\begin{array}{c} \Delta \mu_{\mathrm{nonpolar}}\approx pV+\gamma A+\Delta \mu^{\mathrm{vdW}} \end{array}$$

where *P* is pressure, *V* the solute volume, *γ* the surface tension of water, *A* the solvent-accessible surface area of solute, and Δ*μ*^vdW^ the van der Waals contribution. The surface tension of water at 300 K was set to 0.1032 kcal mol^−1^ Å^−2^ (71.73 mN/m), following Cooper and Dooley (1994). As the van der Waals term contributes <0.1% (Lee and Olson 2013), it was disregarded.

The solute entropy was estimated as a relative vibrational entropy based on statistical mechanics, employing the quasiharmonic approximation applied to the covariance matrix of Cα atomic fluctuations. Prior to calculation, translational and rotational motions were removed, and vibrational modes were extracted utilizing the gmx covar and gmx anaeig utilities. Contributions from translational and rotational entropy were disregarded due to their negligible pressure dependence (Wakai et al. 2014). The entropy difference in vacuo was derived from molecular dynamics snapshots in solution, under the assumption that solvation does not induce structural alterations between states A and H.

## Supplementary Material

evag068_Supplementary_Data

## Data Availability

The C++ implementation of the codon substitution model, developed through modifications of the EvoRadical program, is available at https://github.com/RadicalHTTH/radicalHTTH.git.
