# Supplementary material for: Pressure-tolerant Evolution in Rhodopsin of Deep-diving Whales
Source: Genome Biol Evol. 2026 Mar 16;18(4):evag068. doi: 10.1093/gbe/evag068 (PMC13089582; doi:10.1093/gbe/evag068)
Supplement: evag068_Supplementary_Data [file evag068_supplementary_data.docx]

**Supplementary Table 1** List of genome assemblies used in this study.

| **Scientific name** | **Assembly ID** | **Citation** |
| --- | --- | --- |
| *Phocoena phocoena* | GCA_003071005.2 | Autenrieth et al. 2018 |
| *Phocoena sinus* | GCA_008692025.1 | Morin et al. 2021 |
| *Neophocaena sunameri* | GCA_026225855.1 | Yin et al. 2022 |
| *Monodon monoceros* | GCA_005125345.1 | Westbury et al. 2019 |
| *Delphinapterus leucas* | GCA_002288925.3 | Jones et al. 2017 |
| *Tursiops truncatus* | GCA_001922835.1 | Zoonomia Consortium 2020 |
| *Tursiops aduncus* | GCA_003227395.1 | Vijay et al. 2018 |
| *Delphinus delphis* | GCA_949987515.2 | Ding et al. 2024 |
| *Stenella coeruleoalba* | GCA_951394435.1 | Davison et al. 2024a |
| *Stenella clymene* | Stenella_clymene | DNA Zoo* |
| *Stenella frontalis* | Stenella_frontalis | Dudchenko et al. 2017 |
| *Sousa chinensis* | GCA_003521335.2 | Ming et al. 2019 |
| *Stenella attenuata* | Stenella_attenuata | DNA Zoo |
| *Stenella longirostris orientalis* | Stenella_longirostris_orientalis | DNA Zoo |
| *Steno bredanensis* | GCA_028646385.1 | DNA Zoo |
| *Grampus griseus* | GCA_030267925.1 | DNA Zoo |
| *Pseudorca crassidens* | GCA_039906515.1 | VGP (May 31, 2024) |
| *Peponocephala electra* | Peponocephala_electra | DNA Zoo |
| *Globicephala melas* | GCA_006547405.1 | DNA Zoo |
| *Leucopleurus acutus* | GCA_964270905.1 | DToL (Sep 29, 2024) |
| *Lagenorhynchus albirostris* | GCA_949774975.1 | Davison et al. 2024b |
| *Aethalodelphis obliquidens* | GCA_003676395.1 | DNA Zoo |
| *Cephalorhynchus commersonii* | Cephalorhynchus_commersonii | DNA Zoo |
| *Orcinus orca* | GCA_937001465.1 | Foote et al. 2022 |
| *Inia geoffrensis* | GCA_004363515.1 | Zoonomia Consortium 2020 |
| *Lipotes vexillifer* | GCA_000442215.2 | Zhou et al. 2013 |
| *Mesoplodon stejnegeri* | Mesoplodon_stejnegeri | DNA Zoo |
| *Mesoplodon densirostris* | GCA_025265405.1 | VGP (Sep 19, 2022) |
| *Mesoplodon europaeus* | Mesoplodon_europaeus | DNA Zoo |
| *Mesoplodon mirus* | GCA_964341445.1 | DToL (Nov 28, 2024) |
| *Mesoplodon bidens* | GCA_004027085.1 | Zoonomia Consortium 2020 |
| *Hyperoodon ampullatus* | GCA_949752795.1 | Feyrer et al. 2024 |
| *Ziphius cavirostris* | GCA_004364475.1 | Zoonomia Consortium 2020 |
| *Platanista minor* | GCA_004363435.1 | Zoonomia Consortium 2020 |
| *Platanista gangetica* | PRJNA1206299 | Sharma et al. 2025 |
| *Kogia breviceps* | GCA_026419965.1 | VGP (Nov 27, 2022) |
| *Kogia sima* | CNP0000758/CNS0152180 | Yuan et al. 2021 |
| *Physeter macrocephalus* | GCA_002837175.5 | Fan et al. 2019 |
| *Balaenoptera musculus* | GCA_009873245.3 | Bukhman et al. 2024 |
| *Balaenoptera borealis* | GCA_965194805.1 | DToL (Mar 10, 2025) |
| *Balaenoptera edeni* | GCA_052818205.1 | Lin et al. 2025 |
| *Balaenoptera ricei* | GCA_028023285.1 | VGP (Jan 30, 2023) |
| *Balaenoptera physalus* | GCA_023338255.1 | Wolf et al. 2022 |
| *Megaptera novaeangliae* | GCA_041834305.1 | Carminati et al. 2024 |
| *Eschrichtius robustus* | GCA_028021215.1 | VGP (Jan 31, 2023) |
| *Balaenoptera acutorostrata* | GCA_949987535.1 | Brownlow et al. 2024 |
| *Balaenoptera bonaerensis* | GCA_000978805.1 | Kishida et al. 2015 |
| *Caperea marginata* | GCA_029224305.1 | Wolf et al. 2023 |
| *Eubalaena glacialis* | GCA_028564815.2 | VGP (Feb 9, 2023) |
| *Eubalaena australis* | RWref | DNA Zoo |
| *Balaena mysticetus* | The Bowhead Whale Genome Resource | Keane et al. 2015 |
| *Hippopotamus amphibius* | GCA_004027065.2 | Zoonomia Consortium 2020 |

* For the sources of genome data obtained from DNA Zoo, see Dudchenko et al. (2017), Dudchenko et al. (2018), and the Acknowledgements in the main text.

**Supplementary Table 2** Top p% of partition (p), log-likelihood ($\mathcal{l}$), AIC, and estimated parameters (*κ*, *ω*, *γ*) in the branch model.

| Model | p | $\mathcal{l}$ | AIC | *κ* | *ω* | *γ* |
| --- | --- | --- | --- | --- | --- | --- |
| Alternative | 10 | −3132.479 | 6474.958 | 5.357 | 0.302 | *γ*_fg_=4.088, *γ*_bg_=0.696 |
|  | 20 | −3127.315 | 6464.630 | 5.806 | 0.376 | *γ*_fg_=1.177, *γ*_bg_=0.385 |
|  | 30 | −3118.961 | 6447.922 | 6.431 | 0.482 | *γ*_fg_=0.778, *γ*_bg_=0.293 |
|  | 40 | −3110.501 | 6431.002 | 6.257 | 0.559 | *γ*_fg_=0.601, *γ*_bg_=0.227 |
|  | 50 | −3105.313 | 6420.626 | 6.107 | 0.622 | *γ*_fg_=0.492, *γ*_bg_=0.201 |
|  | 60 | −3104.646 | 6419.292 | 6.044 | 0.769 | *γ*_fg_=0.415, *γ*_bg_=0.201 |
|  | 70 | −3111.006 | 6432.012 | 5.409 | 0.804 | *γ*_fg_=0.551, *γ*_bg_=0.225 |
|  | 80 | −3132.127 | 6474.254 | 5.353 | 0.325 | *γ*_fg_=2.950, *γ*_bg_=0.827 |
|  | 90 | −3132.977 | 6475.953 | 5.359 | 0.289 | *γ*_fg_=2.892, *γ*_bg_=0.954 |
| Null | 10 | −3139.263 | 6486.527 | 5.352 | 0.301 | 0.920 |
|  | 20 | −3129.964 | 6467.927 | 5.803 | 0.375 | 0.438 |
|  | 30 | −3126.206 | 6460.412 | 6.445 | 0.480 | 0.327 |
|  | 40 | −3121.174 | 6450.349 | 6.324 | 0.561 | 0.249 |
|  | 50 | −3110.342 | 6428.684 | 6.112 | 0.620 | 0.220 |
|  | 60 | −3109.448 | 6426.896 | 6.048 | 0.766 | 0.215 |
|  | 70 | −3120.393 | 6448.786 | 5.430 | 0.802 | 0.245 |
|  | 80 | −3139.278 | 6486.556 | 5.358 | 0.322 | 0.914 |
|  | 90 | −3141.118 | 6490.235 | 5.384 | 0.291 | 1.027 |

**Supplementary Table 3** Top p% of partition (p), log-likelihood ($\mathcal{l}$), AIC, and posterior probability of belonging to each site class (i)–(iv) in the site model.

| Model | p | $\mathcal{l}$ | AIC | (i) | (ii) | (iii) | (iv) |
| --- | --- | --- | --- | --- | --- | --- | --- |
| Alternative | 10 | −3068.069 | 6354.137 | 0.593 | 0.048 | 0.337 | 0.022 |
|  | 20 | −3066.815 | 6351.630 | 0.589 | 0.132 | 0.210 | 0.069 |
|  | 30 | −3054.982 | 6327.965 | 0.579 | 0.144 | 0.205 | 0.072 |
|  | 40 | −3047.221 | 6312.441 | 0.597 | 0.131 | 0.201 | 0.071 |
|  | 50 | −3037.306 | 6292.613 | 0.587 | 0.124 | 0.225 | 0.064 |
|  | 60 | −3036.982 | 6291.963 | 0.554 | 0.177 | 0.176 | 0.093 |
|  | 70 | −3069.230 | 6356.460 | 0.584 | 0.155 | 0.161 | 0.100 |
|  | 80 | −3085.563 | 6389.127 | 0.551 | 0.289 | 0.096 | 0.064 |
|  | 90 | −3094.245 | 6406.490 | 0.615 | 0.000 | 0.000 | 0.385 |
| Null | 10 | −3096.402 | 6404.804 | 0.685 | NA | 0.315 | NA |
|  | 20 | −3084.415 | 6380.829 | 0.704 | NA | 0.296 | NA |
|  | 30 | −3083.274 | 6378.549 | 0.738 | NA | 0.262 | NA |
|  | 40 | −3070.790 | 6353.580 | 0.701 | NA | 0.299 | NA |
|  | 50 | −3061.181 | 6334.362 | 0.710 | NA | 0.290 | NA |
|  | 60 | −3065.659 | 6343.318 | 0.761 | NA | 0.239 | NA |
|  | 70 | −3085.515 | 6383.030 | 0.701 | NA | 0.299 | NA |
|  | 80 | −3133.414 | 6478.827 | 0.687 | NA | 0.313 | NA |
|  | 90 | −3136.889 | 6485.778 | 1.000 | NA | 0.000 | NA |

**Supplementary Table 4** List of codon sites with a posterior probability exceeding 0.95 for assignment to site classes with γ > 1 in the site model.

| Site | (i) | (ii) | (iii) | (iv) |
| --- | --- | --- | --- | --- |
| 7 | 0.000 | 0.870 | 0.000 | 0.130 |
| 16 | 0.000 | 0.000 | 0.002 | 0.998 |
| 24 | 0.001 | 0.743 | 0.000 | 0.256 |
| 45 | 0.001 | 0.745 | 0.000 | 0.254 |
| 51 | 0.001 | 0.706 | 0.000 | 0.293 |
| 83 | 0.000 | 0.982 | 0.000 | 0.018 |
| 88 | 0.000 | 0.998 | 0.000 | 0.002 |
| 94 | 0.001 | 0.895 | 0.000 | 0.105 |
| 101 | 0.002 | 0.830 | 0.000 | 0.168 |
| 105 | 0.003 | 0.853 | 0.000 | 0.143 |
| 112 | 0.000 | 0.000 | 0.001 | 0.999 |
| 158 | 0.002 | 0.810 | 0.000 | 0.188 |
| 169 | 0.002 | 0.828 | 0.000 | 0.170 |
| 194 | 0.000 | 0.856 | 0.000 | 0.144 |
| 195 | 0.000 | 0.024 | 0.000 | 0.976 |
| 213 | 0.000 | 0.843 | 0.000 | 0.157 |
| 220 | 0.001 | 0.749 | 0.000 | 0.250 |
| 270 | 0.003 | 0.929 | 0.000 | 0.068 |
| 282 | 0.000 | 0.988 | 0.000 | 0.012 |
| 292 | 0.000 | 0.995 | 0.000 | 0.005 |
| 299 | 0.000 | 0.974 | 0.000 | 0.026 |
| 325 | 0.001 | 0.745 | 0.000 | 0.254 |
| 329 | 0.000 | 0.840 | 0.000 | 0.160 |
| 333 | 0.000 | 0.975 | 0.000 | 0.025 |

**Supplementary Table 5** Mean ± standard deviation (SD) differences in isothermal compressibility ($\kappa_{T}$) between atmospheric (0.1 MPa) and high-pressure (30 MPa) conditions in the A/S299^7.46a^ reciprocal mutants.

| Species/Lineage | A299^7.46a^ [GPa⁻¹] | S299^7.46a^ [GPa⁻¹] |
| --- | --- | --- |
| *Ziphius cavirostris* | −9.745e−02 ± 6.727e−02 | 1.467e−01 ± 2.801e−02 |
| *Phocoena phocoena* | −4.039e−02 ± 2.303e−02 | 2.945e−02 ± 4.259e−02 |
| *Caperea marginata* | −6.366e−02 ± 8.104e−02 | 2.256e−01 ± 5.659e−02 |
| *Lipotes vexillifer* | 6.933e−02 ± 3.047e−02 | 1.962e−01 ± 4.431e−02 |
| Ancestral node of  Physeteroidea | −3.055e−01 ± 1.567e−01 | 5.426e−02 ± 2.424e−02 |
| Ancestral node of  Ziphiidae | −1.935e−01 ± 4.411e−02 | 1.535e−01 ± 3.521e−02 |

**Supplementary Table 6** Mean ± standard deviation (SD) differences in mean RMSF across helices I–VIII between atmospheric (0.1 MPa) and high-pressure (30 MPa) conditions in the A/S299^7.46a^ reciprocal mutants.

| Species/Lineage | A299^7.46a^ [Å] | S299^7.46a^ [Å] |
| --- | --- | --- |
| *Ziphius cavirostris* | −2.604e−02 ± 2.939e−01 | 7.561e−01 ± 4.711e−01 |
| *Phocoena phocoena* | −8.985e−02 ± 4.310e−01 | 1.329e−01 ± 5.816e−01 |
| *Caperea marginata* | 1.551e−01 ± 2.891e−01 | 2.469e−01 ± 2.651e−01 |
| *Lipotes vexillifer* | −3.342e−01 ± 2.479e−01 | 4.183e−01 ± 5.313e−01 |
| Ancestral node of  Physeteroidea | −3.817e−02 ± 4.789e−01 | 5.160e−02 ± 3.895e−01 |
| Ancestral node of  Ziphiidae | −3.817e−02 ± 4.789e−01 | 1.096e−00 ± 7.005e−01 |

**Supplementary Table 7** Mean ± standard deviation (SD) in the pressure-dependent free-energy shift of A/S299^7.46a^ rhodopsin variants.

| Species/Lineage | Energy | A299^7.46a^ [kcal mol^-1^] | S299^7.46a^ [kcal mol^-1^] |
| --- | --- | --- | --- |
| *Ziphius cavirostris* | Δ*G_p_* | 243 ± 6 | 346 ± 6 |
|  | Δ*E*_conf_ | −56 ± 3 | 98 ± 3 |
|  | −*T*Δ*S* | 14 ± 1 | −26 ± 0 |
|  | ΔΔ*μ* | 285 ± 2 | 274 ± 3 |
| *Phocoena phocoena* | Δ*G_p_* | 208 ± 21 | 252 ± 12 |
|  | Δ*E*_conf_ | −66 ± 8 | 2 ± 6 |
|  | −*T*Δ*S* | 17 ± 2 | −1 ± 1 |
|  | ΔΔ*μ* | 257 ± 11 | 251 ± 5 |
| *Caperea marginata* | Δ*G_p_* | 401 ± 10 | 445 ± 16 |
|  | Δ*E*_conf_ | 21 ± 6 | 145 ± 6 |
|  | −*T*Δ*S* | −5 ± 2 | −25 ± 1 |
|  | ΔΔ*μ* | 385 ± 2 | 325 ± 9 |
| *Lipotes vexillifer* | Δ*G_p_* | 268 ± 13 | 410 ± 19 |
|  | Δ*E*_conf_ | −22 ± 8 | 55 ± 4 |
|  | −*T*Δ*S* | 13 ± 1 | −28 ± 1 |
|  | ΔΔ*μ* | 277 ± 4 | 383 ± 14 |
| Ancestral node of  Physeteroidea | Δ*G_p_* | 229 ± 15 | 353 ± 16 |
|  | Δ*E*_conf_ | −60 ± 6 | −6 ± 7 |
|  | −*T*Δ*S* | 13 ± 2 | 4 ± 2 |
|  | ΔΔ*μ* | 276 ± 7 | 355 ± 7 |
| Ancestral node of  Ziphiidae | Δ*G_p_* | 256 ± 16 | 531 ± 6 |
|  | Δ*E*_conf_ | −60 ± 4 | 127 ± 5 |
|  | −*T*Δ*S* | 13 ± 2 | −24 ± 0 |
|  | ΔΔ*μ* | 303 ± 10 | 428 ± 1 |


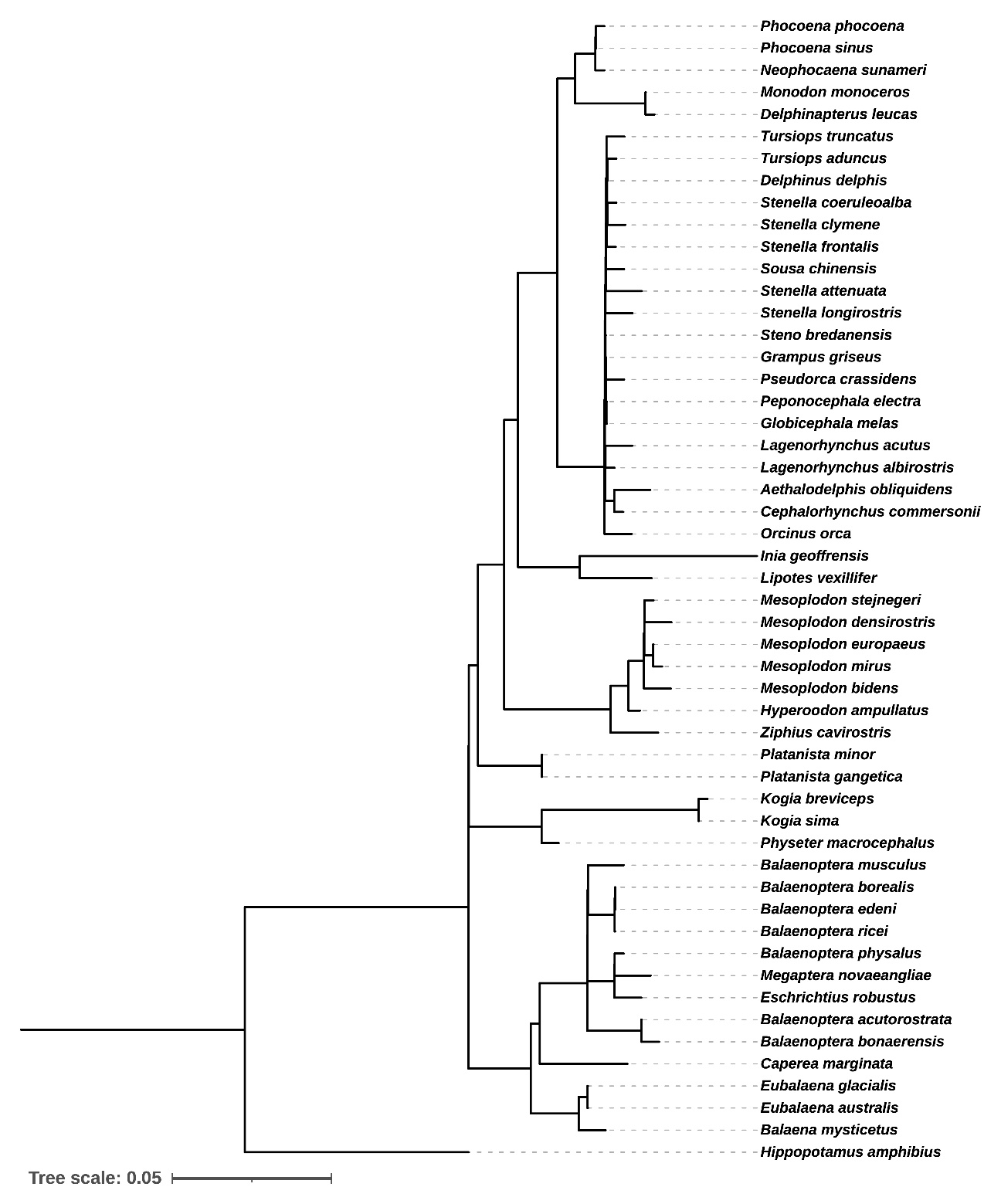
**Supplementary FIG. 1.** The phylogenetic tree inferred under the best-fitting partition model (the model defining radical substitutions as those in the top 60% of |Δ*K⁰*|).

**
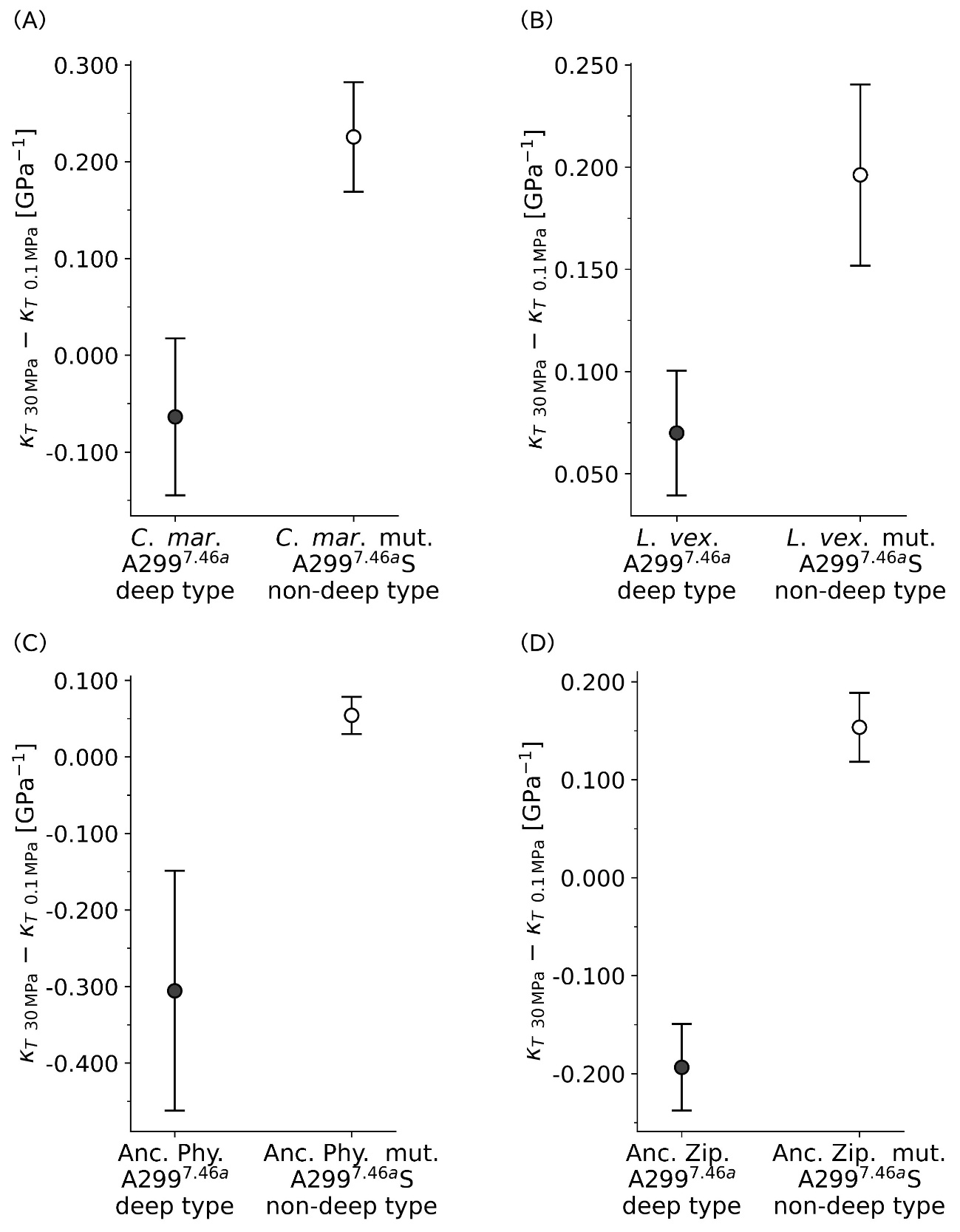
Supplementary FIG. 2.** Differences in isothermal compressibility ($\kappa_{T}$) between atmospheric (0.1 MPa) and high-pressure (30 MPa) conditions are shown as the mean ± standard deviation (SD). (A) *Caperea marginata* wild-type (A299, deep-diving type) and mutant (A299S, non-deep-diving type). (B) *Lipotes vexillifer* wild-type (A299, deep-diving type) and mutant (A299S, non-deep-diving type). (C) wild-type of reconstructed ancestral node leading to Physeteroidea (A299, deep-diving type) and mutant (A299S, non-deep-diving type). (D) wild-type of reconstructed ancestral node leading to Ziphiidae (A299, deep-diving type) and mutant (A299S, non-deep-diving type). The points represent the mean values, whereas vertical bars indicate the SD. The black-filled symbol denotes the deep-diving type (A299), and the open symbol denotes the non-deep-diving type (S299).

**
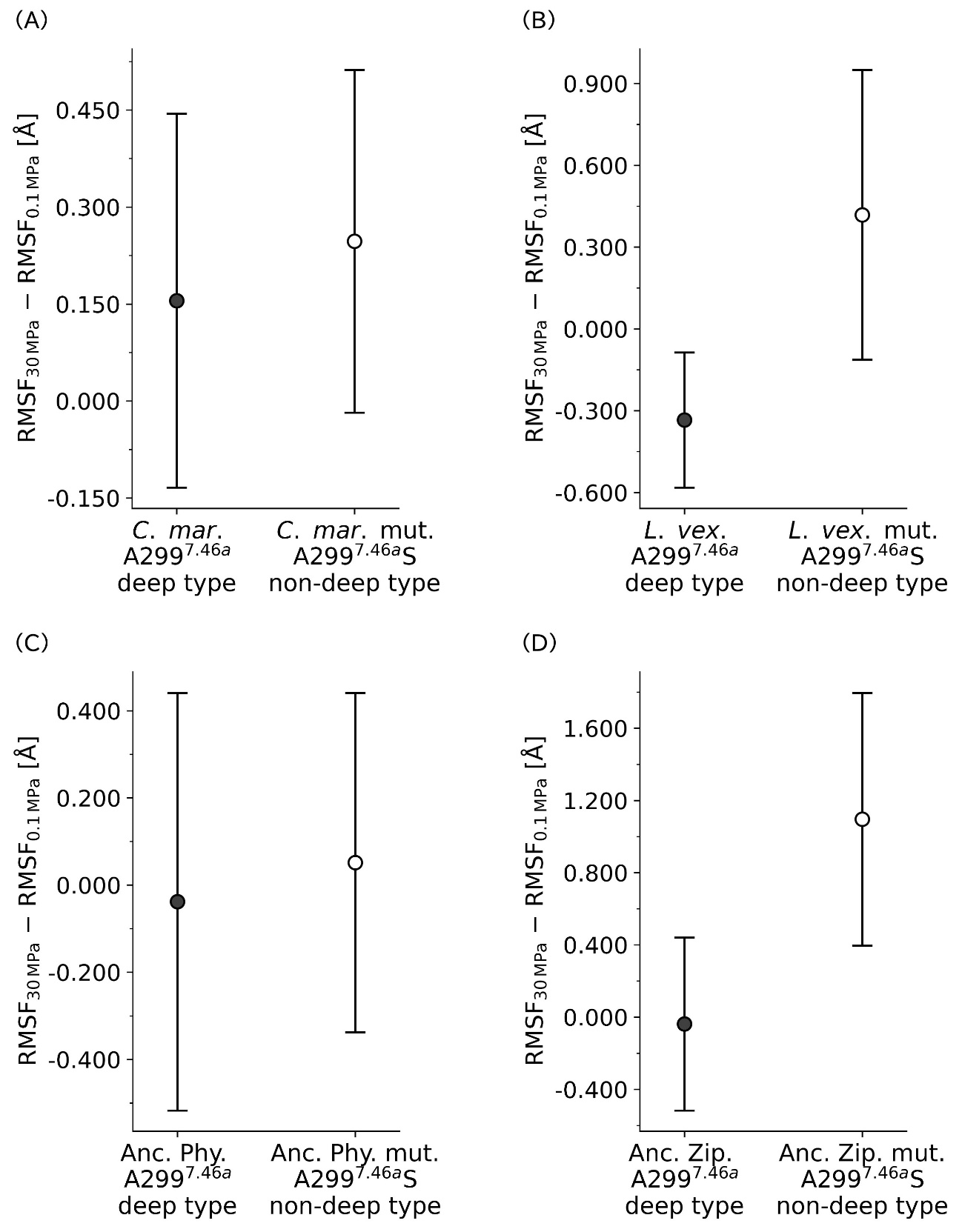
Supplementary FIG. 3.** Mean RMSF differences ($\mathrm{RMS}F_{30 \mathrm{MPa}}-\mathrm{RMS}F_{0.1 \mathrm{MPa}}$) across helices I–VIII for wild-type and mutant rhodopsins as the mean ± standard deviation (SD). (A) *Caperea marginata* wild-type (A299, deep-diving type) and mutant (A299S, non-deep-diving type). (B) *Lipotes vexillifer* wild-type (A299, deep-diving type) and mutant (A299S, non-deep-diving type). (C) wild-type of reconstructed ancestral node leading to Physeteroidea (A299, deep-diving type) and mutant (A299S, non-deep-diving type). (D) wild-type of reconstructed ancestral node leading to Ziphiidae (A299, deep-diving type) and mutant (A299S, non-deep-diving type). The black symbols indicate deep-diving types, and the open symbols indicate non-deep-diving types.

**
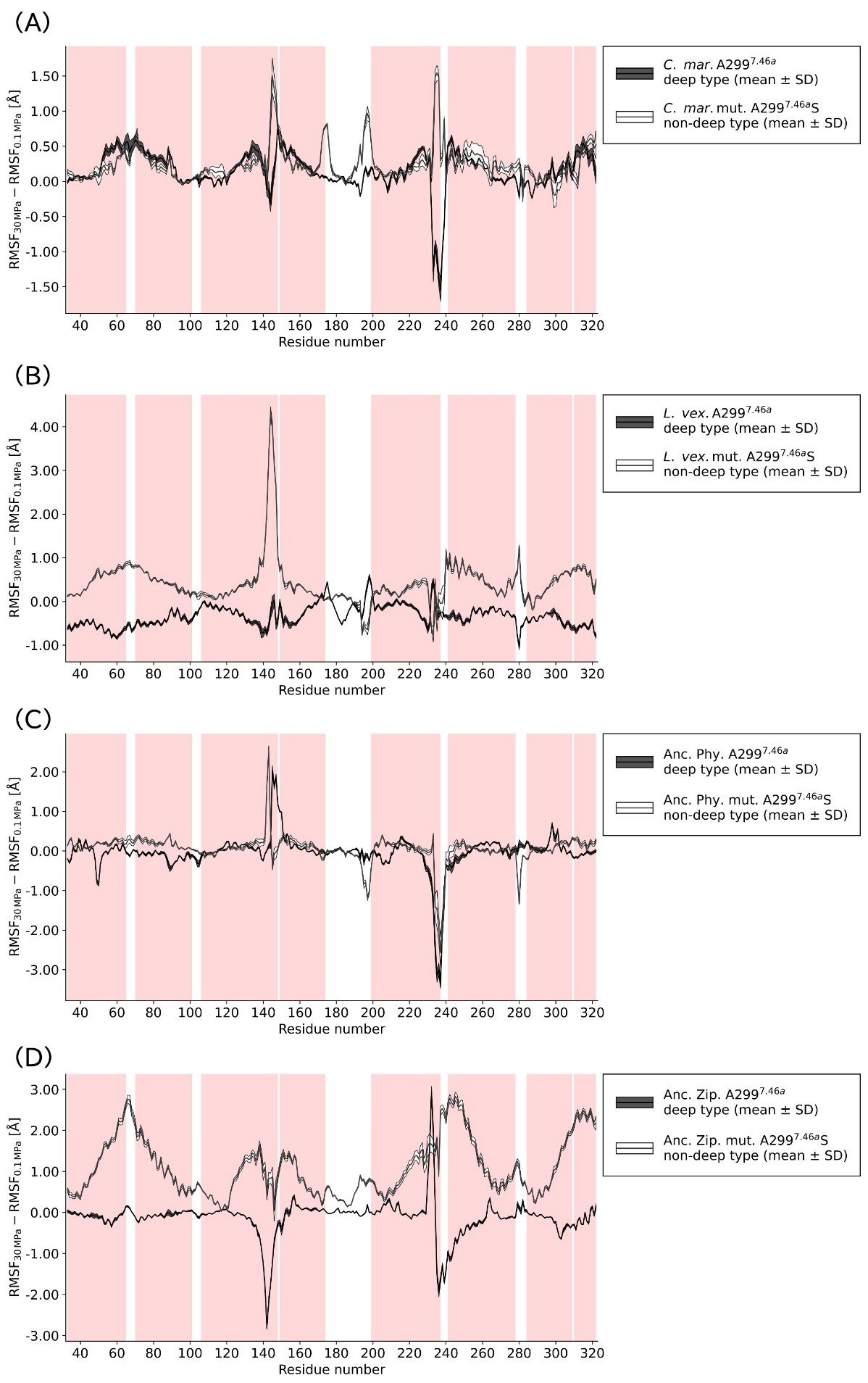
Supplementary FIG. 4.** Residue-level RMSF differences along helices I–VIII. Pink shading indicates helical regions. Black solid lines represent mean values, and the shaded areas indicate ± SD. The black shaded areas indicate deep-diving types, and the open shaded areas indicate non-deep-diving types. (A) *Caperea marginata* wild-type (A299, deep-diving type) and mutant (A299S, non-deep-diving type). (B) *Lipotes vexillifer* wild-type (A299, deep-diving type) and mutant (A299S, non-deep-diving type). (C) wild-type of reconstructed ancestral node leading to Physeteroidea (A299, deep-diving type) and mutant (A299S, non-deep-diving type). (D) wild-type of reconstructed ancestral node leading to Ziphiidae (A299, deep-diving type) and mutant (A299S, non-deep-diving type).


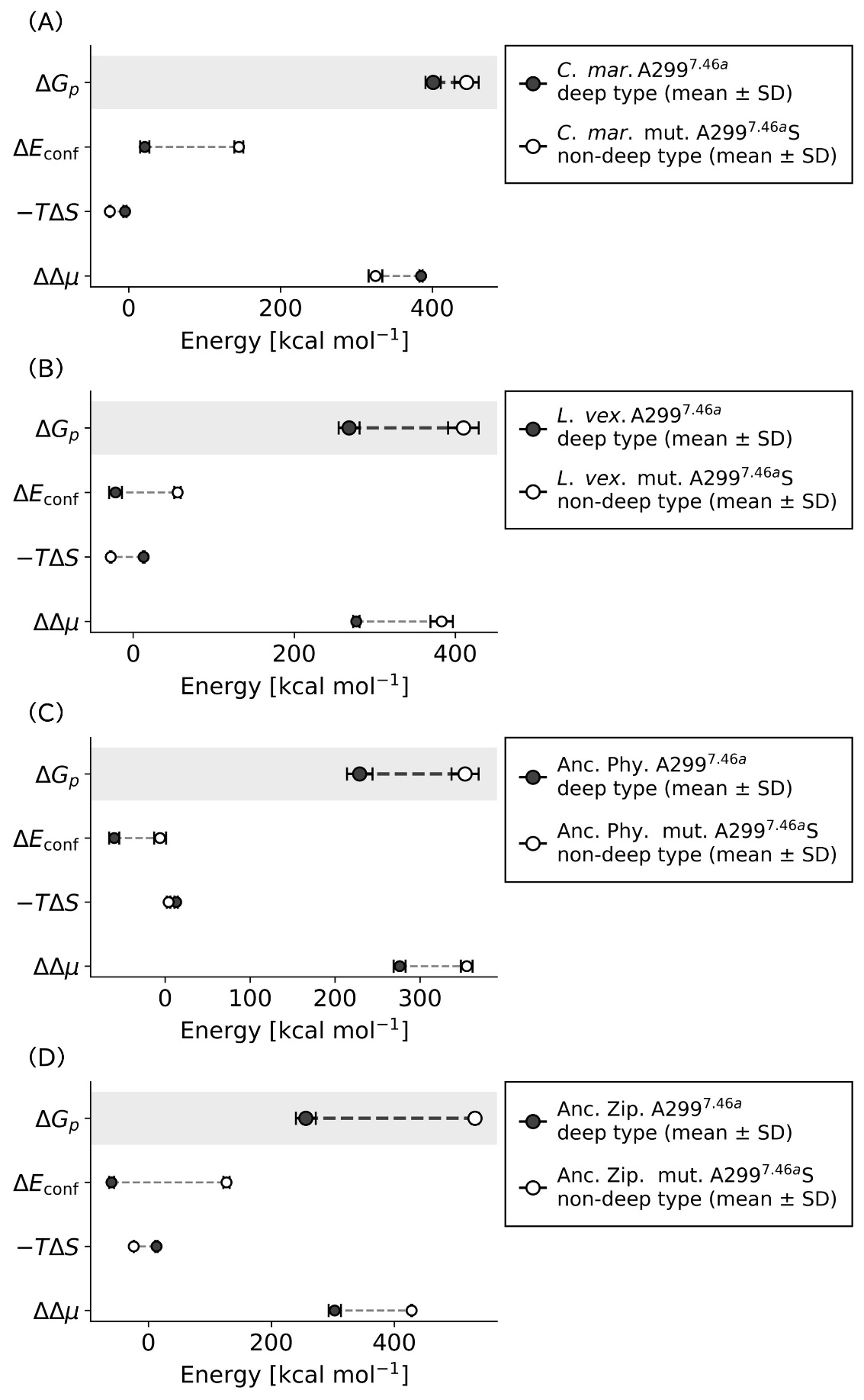
**Supplementary FIG. 5.** The pressure-dependent free-energy shift of A/S299 rhodopsin variants in (A) *Caperea marginata*, (B) *Lipotes vexillifer*, (C) ancestral node of Physeteroidea, (D) ancestral node of Ziphiidae. These dumbbell plots summarize the calculated free-energy shift components (mean ± SD, kcal mol⁻¹) for wild-type and mutant rhodopsins. The top row in each panel highlights the overall pressure-dependent free-energy change (Δ*G_p_*). In contrast, the lower rows display the conformational energy shift (Δ*E*_conf_), the entropic shift term (−*T*Δ*S*), and the solvation free energy shift contribution (ΔΔ*μ*). Filled black circles indicate the deep-diving types, and open circles represent the non-deep-diving type.

**Literature Cited**

Autenrieth M, et al. 2018. High‐quality whole‐genome sequence of an abundant Holarctic odontocete, the harbour porpoise (*Phocoena phocoena*). Mol Ecol Resour. 18(6):1469-1481. https://doi.org/10.1111/1755-0998.12932.

Brownlow A, Davison NJ, Morin PA, Darwin Tree of Life Consortium. 2024. The genome sequence of the minke whale, *Balaenoptera acutorostrata* Lacépède, 1804. Wellcome Open Res. 9:706.
https://doi.org/10.12688/wellcomeopenres.23367.1.

Bukhman YV, et al. 2024. A high-quality blue whale genome, segmental duplications, and historical demography. Mol Biol Evol. 41(3):msae036.
https://doi.org/10.1093/molbev/msae036.

Carminati MV, et al. 2024. Novel *Megaptera novaeangliae* (Humpback whale) haplotype chromosome-level reference genome. Sci Data. 11(1):1113.
https://doi.org/10.1038/s41597-024-03922-9.

Culibrk L, et al. "The genome of the Pilot Whale (Globicephala melas)." Unpublished. https://www.ncbi.nlm.nih.gov/nuccore/SWEB00000000.1.

Culibrk L, et al. "The genome of the whitesided dolphin." Unpublished. https://www.ncbi.nlm.nih.gov/nuccore/RCWK00000000.1.

Davison NJ, Morin PA, Darwin Tree of Life Consortium. 2024a. The genome sequence of the striped dolphin, *Stenella coeruleoalba* (Meyen, 1833). Wellcome Open Res. 9:727. https://doi.org/10.12688/wellcomeopenres.23374.1.

Davison NJ, Morin P, Darwin Tree of Life Consortium. 2024b. The genome sequence of the white-beaked dolphin, Lagenorhynchus albirostris (Gray, 1846). Wellcome Open Res. 9:687. https://doi.org/10.12688/wellcomeopenres.23369.1.

Ding K, et al. 2024. Chromosome-level genome provides insights into environmental adaptability and innate immunity in the common dolphin (*Delphinus delphis*). BMC genomics. 25(1):373. https://doi.org/10.1186/s12864-024-10268-4.

Dudchenko O, et al. 2017. De novo assembly of the Aedes aegypti genome using Hi-C yields chromosome-length scaffolds. Science. 356(6333):92-95.
https://doi.org/10.1126/science.aal3327.

Dudchenko O, et al. 2018. The Juicebox Assembly Tools module facilitates de novo assembly of mammalian genomes with chromosome-length scaffolds for under $1000. BioRxiv. 254797. https://doi.org/10.1101/254797.

Fan G, et al. 2019. The first chromosome‐level genome for a marine mammal as a resource to study ecology and evolution. Mol Ecol Resour. 19(4):944-956. https://doi.org/10.1111/1755-0998.13003.

Feyrer LJ, de Greef E, Darwin Tree of Life Consortium. 2024. The genome sequence of the Northern Bottlenose Whale, *Hyperoodon ampullatus* (Forster, 1770). Wellcome Open Res. 9:410.
https://doi.org/10.12688/wellcomeopenres.22743.1.

Foote A, Bunskoek P, Darwin Tree of Life Consortium. 2022. The genome sequence of the killer whale, *Orcinus orca* (Linnaeus, 1758). Wellcome Open Res. 7:250. https://doi.org/10.12688/wellcomeopenres.18278.1.

Jones SJ, et al. 2017. The genome of the beluga whale (*Delphinapterus leucas*). Genes. 8(12):378. https://doi.org/10.3390/genes8120378.

Keane M, et al. 2015. Insights into the evolution of longevity from the bowhead whale genome. Cell Rep. 10(1):112-122. https://doi.org/10.1016/j.celrep.2014.12.008.

Kishida T, Thewissen JGM, Hayakawa T, Imai H, Agata K. 2015. Aquatic adaptation and the evolution of smell and taste in whales. Zool Lett. 1(1):9.
https://doi.org/10.1186/s40851-014-0002-z.

Lin YT, et al. 2025. Chromosome-Level Genome Assembly of Eden's Whale Clarifies the Taxonomy and Speciation of Bryde's Whale Complex. Mol Biol Evol. 42(10):msaf234. https://doi.org/10.1093/molbev/msaf234.

Ming Y, et al. 2019. Molecular footprints of inshore aquatic adaptation in Indo-Pacific humpback dolphin (*Sousa chinensis*). Genomics. 111(5):1034-1042. https://doi.org/10.1016/j.ygeno.2018.07.015.

Morin PA, et al. 2021. Reference genome and demographic history of the most endangered marine mammal, the vaquita. Mol Ecol Resour. 21(4):1008-1020. https://doi.org/10.1111/1755-0998.13284.

Sharma SP, et al. 2025. Genome assembly of Gangetic dolphin (*Platanista gangetica*) reveals signature of regressive evolution. Genome Biol Evol. evaf227. https://doi.org/10.1093/gbe/evaf227.

Vijay N, et al. 2018. Population genomic analysis reveals contrasting demographic changes of two closely related dolphin species in the last glacial. Mol Biol Evol. 35(8):2026-2033. https://doi.org/10.1093/molbev/msy108.

Westbury MV, Petersen B, Garde E, Heide-Jørgensen MP, Lorenzen ED. 2019. Narwhal genome reveals long-term low genetic diversity despite current large abundance size. IScience. 15:592-599.
https://doi.org/10.1016/j.isci.2019.03.023.

Wolf M, De Jong M, Halldórsson SD, Árnason Ú, Janke A. 2022. Genomic impact of whaling in North Atlantic fin whales. Mol Biol Evol. 39(5):msac094. https://doi.org/10.1093/molbev/msac094.

Wolf M, et al. 2023. The genome of the pygmy right whale illuminates the evolution of rorquals. BMC Biol. 21(1):79. https://doi.org/10.1186/s12915-023-01579-1.

Yin D, et al. 2022. Gapless genome assembly of East Asian finless porpoise. Sci Data. 9(1):765. https://doi.org/10.1038/s41597-022-01868-4.

Yuan Y, et al. 2021. Comparative genomics provides insights into the aquatic adaptations of mammals. Proc Natl Acad Sci USA. 118(37):e2106080118.
https://doi.org/10.1073/pnas.2106080118.

Zhou X, et al. 2013. Baiji genomes reveal low genetic variability and new insights into secondary aquatic adaptations. Nat Commun. 4(1):2708.
https://doi.org/10.1038/ncomms3708.

Zoonomia Consortium. 2020. A comparative genomics multitool for scientific discovery and conservation. Nature. 587.7833: 240-245.
https://doi.org/10.1038/s41586-020-2876-6.
